# Paradoxical self-sustained dynamics emerge from orchestrated excitatory and inhibitory homeostatic plasticity rules

**DOI:** 10.1073/pnas.2200621119

**Published:** 2022-10-17

**Authors:** Saray Soldado-Magraner, Michael J. Seay, Rodrigo Laje, Dean V. Buonomano

**Affiliations:** ^a^Department of Neurobiology, University of California, Los Angeles, CA 90095;; ^b^Departamento de Ciencia y Tecnología, Universidad Nacional de Quilmes, Bernal, B1876BXD Argentina;; ^c^Consejo Nacional de Investigaciones Científicas y Técnicas (CONICET), Buenos Aires, C1425FQB Argentina;; ^d^Department of Psychology, University of California, Los Angeles, CA 90095

**Keywords:** inhibition-stabilized networks, paradoxical effect, homeostatic plasticity

## Abstract

Self-sustained neural activity maintained through local recurrent connections is of fundamental importance to cortical function. Converging theoretical and experimental evidence indicates that cortical circuits generating self-sustained dynamics operate in an inhibition-stabilized regime. Theoretical work has established that four sets of weights (*W_E←E_*, *W_E←I_*, *W_I←E_*, and *W_I←I_*) must obey specific relationships to produce inhibition-stabilized dynamics, but it is not known how the brain can appropriately set the values of all four weight classes in an unsupervised manner to be in the inhibition-stabilized regime. We prove that standard homeostatic plasticity rules are generally unable to generate inhibition-stabilized dynamics and that their instability is caused by a signature property of inhibition-stabilized networks: the paradoxical effect. In contrast, we show that a family of “cross-homeostatic” rules overcome the paradoxical effect and robustly lead to the emergence of stable dynamics. This work provides a model of how—beginning from a silent network—self-sustained inhibition-stabilized dynamics can emerge from learning rules governing all four synaptic weight classes in an orchestrated manner.

## Significance

Cortical networks have the remarkable ability to self-assemble into dynamic regimes in which excitatory positive feedback is balanced by recurrent inhibition. This inhibition-stabilized regime is increasingly viewed as the default dynamic regime of the cortex, but how it emerges in an unsupervised manner remains unknown. We prove that classic forms of homeostatic plasticity are unable to drive recurrent networks to an inhibition-stabilized regime due to the well-known paradoxical effect. We next derive a novel family of cross-homeostatic rules that lead to the unsupervised emergence of inhibition-stabilized networks. These rules shed new light on how the brain may reach its default dynamic state and provide a valuable tool to self-assemble artificial neural networks into ideal computational regimes.

Self-sustained patterns of neural activity maintained by local recurrent excitation underlie many cortical computations and dynamic regimes, including the persistent activity associated with working memory (1–3), motor control (4, 5), asynchronous states associated with the default cortical dynamic regime (6–10), and up-states (8, 11). Recurrent excitation also has the potential to drive pathological and epileptiform regimes (12, 13). Converging theoretical and experimental evidence indicates that cortical circuits that generate self-sustained dynamics operate in an inhibition-stabilized regime, in which positive feedback is held in check by recurrent inhibition (7, 14–20). There is also evidence that inhibition-stabilized regimes may comprise the default awake cortical dynamic regime (8, 9, 20).

**Fig. 1.**
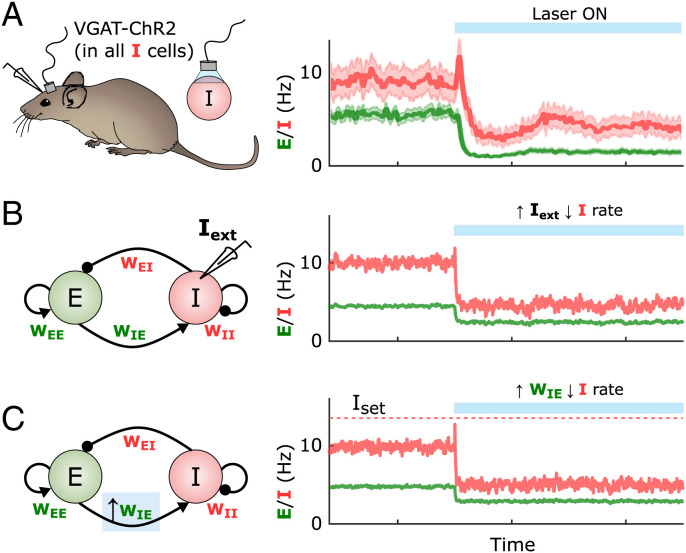
The paradoxical effect in cortical circuits and its implications for plasticity. (*A*) Average inhibitory (red) and excitatory (green) firing rates in the visual cortex of awake mice in the absence of explicit external sensory stimulation. When inhibitory neurons are optogenetically activated, the firing rates of the inhibitory neurons show a paradoxical decrease in activity during the stimulation, indicative of an inhibition-stabilized network. Adapted from Sanzeni et al. (20). (*B*) Two-population firing rate model of self-sustained cortical activity. The dynamics of the excitatory (green) and inhibitory (red) populations are governed by four synaptic weights, *W_E←E_*, *W_E←I_*, *W_I←E_*, and *W_I←I_*. Similar to the experimental case shown in *A*, the model shows the paradoxical effect when the inhibitory population is excited via an external current *I*_ext_ = 7. Weights were initialized to *W_EE_* = 5, *W_EI_* = 1.52, *W_IE_* = 10, and *W_II_* = 2.25. (*C*) As in *B*, if an inhibitory population is firing below its homeostatic setpoint, and one were to increase its excitatory weights according to standard homeostatic rules, the increase in excitatory weights would produce a paradoxical and anti-homeostatic decrease in inhibitory neuron firing. Weights were initialized to *W_EE_* = 5, *W_EI_* = 1.52, *W_IE_* = 10, and *W_II_* = 2.25, with a later increase of *W_IE_* = 12.

At the computational level inhibition-stabilized networks are often modeled as a simplified circuit composed of interconnected excitatory (*E*) and inhibitory (*I*) neural populations with four classes of synaptic weights: *W_E←E_*, *W_E←I_*, *W_I←E_*, and *W_I←I_*. In the inhibition-stabilized regime, recurrent excitation produces positive feedback, which is held in check by rapid inhibition. The dynamics settles into a stable fixed-point attractor and instantiates an inhibition-stabilized network. A signature of the inhibition-stabilized regime is the presence of the paradoxical effect, in which an *increase* in excitatory drive to inhibitory neurons produces a net *decrease* in the firing rate of those same inhibitory neurons (14–16, 18), a phenomenon that has been observed in the awake resting cortex (Fig. 1*A*) (20). Analytical and numerical studies have demonstrated that in order to support inhibition-stabilized dynamics, the four weight classes must obey certain “balanced” relationships; for example, if excitation is too strong, runaway (or saturated) excitation occurs, whereas if inhibition is too strong the activity falls into a quiescent fixed point (6, 7, 14–16, 19). However, in most computational models the set of four weights is determined analytically or through numerical searches—or in a few cases by allowing one or two weights to be plastic while appropriately hardwiring the others. In contrast, experimental studies both in vitro and in vivo have shown that self-sustained activity emerges autonomously during development (21–26), indicating that synaptic plasticity rules are in place to orchestrate the unsupervised emergence of inhibition-stabilized dynamics. Additionally, because the four weight classes have been observed to undergo synaptic plasticity in experimental studies (27–32), here we ask how inhibition-stabilized dynamics might emerge in a self-organizing manner.

One possibility is that standard homeostatic forms of plasticity underlie the emergence of inhibition-stabilized dynamics. Homeostatic plasticity rules generally assume that excitatory weights are regulated in a manner proportional to the difference between some ontogenetically determined setpoint and average neural activity (for both excitatory and inhibitory neurons)—and conversely that inhibitory weights onto excitatory neurons are regulated in the opposite direction (33–38). However, it remains an open question whether homeostatic rules can lead to the self-organized emergence of inhibition-stabilized networks. Here we use computational models and analytical methods to explore families of homeostatic plasticity rules that operate in parallel in all four synapse classes and lead to inhibition-stabilized dynamics. We show that when driving the network toward inhibition-stabilized regimes, standard forms of homeostatic plasticity are stable only in a narrow region of parameter space. Here we prove that this instability arises from the paradoxical effect. Indeed, it can be seen that like increasing external input to an inhibitory neuron, increasing the excitatory weights onto an inhibitory neuron firing below its setpoint produces a paradoxical (and anti-homeostatic) decrease in inhibitory activity (Fig. 1 *B* and *C*). While inhibition-stabilized regimes can operate in the presence or absence of external input, here we focus primarily on “fully self-sustained” activity in the absence of any external tonic input; however, we show that our results also apply when external inputs are present.

We conclude that homeostatic manipulations in the inhibitory population lead to paradoxical outcomes, making the rules unstable in this context. Therefore, homeostatic plasticity rules that aim to bring the network to the relevant dynamic regime of the cerebral cortex must work in paradoxical conditions. We developed a family of homeostatic plasticity rules that include “cross-homeostatic” influences and lead to the unsupervised emergence of fully self-sustained dynamics in the inhibition-stabilized regime in a robust manner. These rules are consistent with experimental data and generate explicit predictions regarding the effects of manipulations of excitatory and inhibitory neurons on synaptic plasticity.

## Results

### Standard Homeostatic Plasticity Rules Cannot Account for the Emergence of Stable Self-Sustained Activity.

We first ask, when starting from a network in a silent regime similar to cortical circuits early in development, whether standard homeostatic rules can drive networks to stable self-sustained dynamics in the absence of any external input. Based on experimental studies we assume that both excitatory and inhibitory neurons exhibit ontogenetically programmed firing rate setpoints (38–42) and ask whether homeostatic plasticity of excitatory and inhibitory weights can drive neurons to these setpoints. Homeostatic plasticity rules are traditionally defined by changes in synaptic weights that are proportional to an “error term” defined by the difference between the setpoint and the neurons’ average activity levels (34–37, 39, 43, 44), for example, Δ*W_E__←__E_* ∝ *E*_set_ *− E*_avg_, where any departure of the excitatory activity *E*_avg_ from the setpoint *E*_set_ would lead to a compensatory correction in the value of the weight *W_E__←__E_*.

**Fig. 2.**
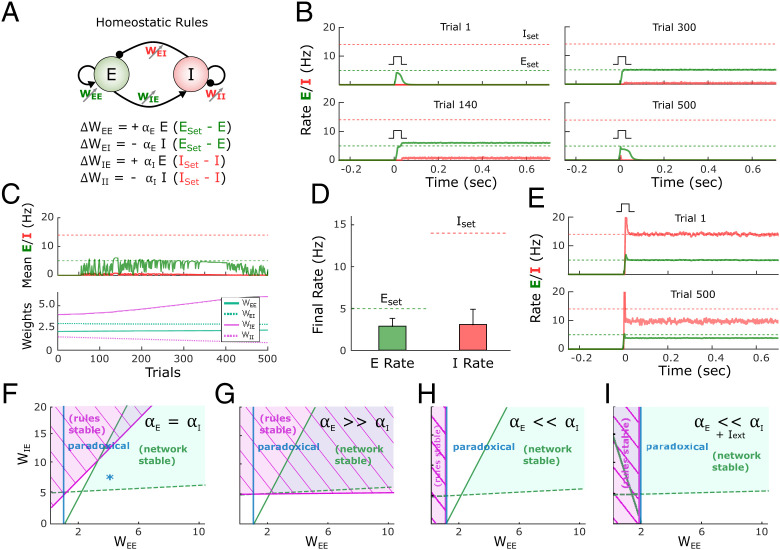
Standard homeostatic rules are stable only in a narrow parameter regime. (*A*) Schematic (*Top*) of the population rate model in which the four weights are governed by a family of homeostatic plasticity rules (*Bottom*). (*B*) Example simulation of the network over the course of simulated development. Each plot shows the firing rate of the excitatory and inhibitory populations over the course of a trial in response to a brief external input (*I*_ex_*_t_* = 7, *I*_dur_ = 10 ms). Note that the pulse is applied on every trial at *t* = 0. *E*_set_ *= 5* and *I*_set_ = 14 represent the target homeostatic setpoints. Weights were initialized to *W_EE_* = 2.1, *W_EI_* = 3, *W_IE_* = 4, and *W_II_* = 2. The learning rate was set to α*_E_* = α*_I_* = 1*e*^−4^. Note that while the network supports self-sustained activity in trial 200, the firing rates do not converge to their setpoints, and by trial 500 the self-sustained dynamics are no longer observed. (*C*) Average rate across trials (Top) for the excitatory and inhibitory populations for the data shown in *B*. Weight dynamics (*Bottom*) produced by the homeostatic rules across trials for the data shown in *B*. (*D*) Average final rate for 100 independent simulations with different weight initializations. Data represent mean ± SEM. (*E*) Simulation of a network starting with the weights sets that generate self-sustained activity at the target setpoints (*E*_set_ = 5 and *I*_set_ = 14 Hz; trial 1, *Top*). After 500 trials the network has diverged from its setpoints, indicating the synaptic plasticity rules are unstable. Weights were initialized to *W_EE_* = 5, *W_EI_* = 1.09, *W_IE_* = 10, and *W_II_* = 1.54. (*F*) Analytical stability regions of the neural and plasticity rule subsystems as a function of the free weights *W_EE_* and *W_IE_*. (Note that once *W_EE_* and *W_IE_* are set to generate self-sustained activity with specific *E*_set_ and *I*_set_ values, *W_EI_* and *W_II_* are fully determined by *W_EE_* and *W_IE_*, respectively). Here the stability plot is obtained by considering equal learning rates for all four plasticity rules (as used for panels *B*–*E*). Blue asterisk corresponds to the initial conditions shown in *E* (*Top*). (*G*) Similar to *F* but with α*_E_* ≫ α*_I_*. (*H*) Similar to *F* but with but with α*_E_* ≪ α*_I_*. To the right of the blue line, the network is in a paradoxical regime (defined by the condition W_EE__*_g_E_ − 1 > 0). (*I*) Condition of stability of the neural system and plasticity rule system when the learning rate on the inhibitory neuron dominates and an external excitatory current is applied to the excitatory neuron. The current produces an enlargement of the stability region of the neural subsystem. Right of blue line shows the area where the network is in a paradoxical regime.

We first examined whether stable self-sustained dynamics can emerge in the standard two-population model (Fig. 1*B*) (19) through homeostatic mechanisms. We initialized the four weights (*W_E__←__E_*, *W_E__←__I_*, *W_I__←__E_*, and *W_I__←__I_*) of the model to small values and applied a standard family of homeostatic plasticity rules to all four weight classes (Fig. 2*A*). It is well established that PV^+^-inhibitory neurons have higher firing rates than pyramidal neurons during periods of self-sustained activity (45, 46); thus, based on previous data using intracellular recordings, we set the setpoints for the *E* and *I* populations to 5 and 14 Hz, respectively (46). We first asked whether the family of four standard homeostatic plasticity rules can lead to a stable self-sustained dynamic regime in response to a brief external input. Since in the absence of external input (or intrinsic spontaneous activity) networks capable of self-sustained activity have a trivial stable silent (down-state) regime, at the beginning of each trial we administered a brief external input to engage the network (low levels of noise were used to avoid fluctuation-induced transitions). Although the rules are homeostatic in nature (e.g., if *I* is below *I*_set_, an increase in *W_I__←__E_* and a decrease in *W_I__←__I_* would be induced), in the example shown in Fig. 2 *B* and *C* the network failed to converge to a stable self-sustained regime (Fig. 2 *B* and *C*). Initially (trial 1) an external input to the excitatory population does not engage recurrent activity because *W_E__←__E_* is too weak. By trial 200 the weights have evolved and the brief external input triggers self-sustained activity, but activities *E* and *I* do not match the corresponding setpoints; the network is in a nonbiologically observed regime in which *E* > *I*, so the weights keep evolving. By trial 600 *E* = *E*_set_ but *I* < *I*_set_, and rather than converging to *I*_set_, the network returns to a regime without self-sustained activity by trial 1,000. At that point both setpoint error terms have increased, leading to continued weight changes (Fig. 2*C*). Results across 100 simulations with different weight initializations (*SI Appendix*, *Supplementary Methods*) further indicate that the standard homeostatic rules are ineffective at driving *E* and *I* toward their respective setpoints and generating stable self-sustained dynamics (Fig. 2*D*).

To gain insights into why a family of homeostatic plasticity rules that might intuitively converge fails to do so, we can consider the case in which a network is initialized to a set of weights that already match *E*_set_ and *I*_set_ (Fig. 2*E*). Although the neural subsystem alone is stable at this condition (trial 1), small fluctuations in *E* and *I* cause the homeostatic rules to drive the weight values and the average activity of the network away from the setpoints (trial 500). It is possible to understand this instability by performing an analytical stability analysis. Specifically, a two-population network in which the weights undergo plasticity can be characterized as a dynamic system composed of two subsystems: the neural subsystem, composed of the two differential equations that define *E* and *I* dynamics, and the synaptic homeostatic plasticity rule subsystem, defined by the four plasticity rules (*SI Appendix*, Section 2.1). We use the two very different time scales of the neural (fast) and plasticity rule (slow) subsystems to perform a quasi-steady-state approximation of the neural subsystem; then we compute the eigenvalues of the four-dimensional plasticity rule subsystem and finally get an analytical expression for the stability condition of the plasticity rules (*SI Appendix*, Section 2.3). For the entire system to be stable, both the neural and plasticity rule subsystems have to be stable. For the results presented in Fig. 2 *B*–*E* we assumed the learning rates driving plasticity onto the excitatory (α*_E_*) and inhibitory neurons (α*_I_*) to be equal. Under these conditions, the standard homeostatic rules are mostly unstable for biologically meaningful parameter values in which the neural system is stable. The regions of stability can be seen in Fig. 2*F*. Critically, Fig. 2*F* shows that the stability region of the neural subsystem, that is, an inhibition-stabilized network (15, 19), is almost entirely within the region where the homeostatic plasticity rule system is unstable. The only region where a stable self-sustained dynamics can exist is the small triangle where the neural and synaptic stability regions overlap (*SI Appendix*, Fig. S1). Only when plasticity onto the excitatory neuron is significantly faster (α*_E_* ≫ α*_I_*, resulting in very slow convergence to the inhibitory setpoints) is there a substantial region of overlap between the stability of the neural and plasticity rule subsystems (Fig. 2*G*; *SI Appendix*, Section 1.1).

Because inhibitory neurons seem to undergo homeostatic plasticity as quickly as or more quickly than excitatory neurons (40–42, 47, 48) we conclude that standard homeostatic rules by themselves do not account for the emergence of stable self-sustained and inhibition-stabilized dynamics. Similarly, a combination of analytical and numerical methods also indicates that variants of these homeostatic rules, such as synaptic scaling, are also stable only in a narrow region of parameter space (*SI Appendix*, Section 1.5). We next show that the inherent instability of standard homeostatic plasticity rules is related to the paradoxical effect.

### The Paradoxical Effect Hampers the Ability of Homeostatic Rules to Lead to Self-Sustained Activity.

The inability of the homeostatic plasticity rules to generate stable self-sustained activity is in part a consequence of the paradoxical effect, a counterintuitive yet well described property of two-population models of inhibition-stabilized networks (14, 15). Specifically, if during self-sustained activity one increases the excitatory drive to the inhibitory population, the net result is a decrease in the firing rate of the inhibitory units. This paradoxical effect can be understood in terms of the *I*→*E*→*I* loop: The increased inhibitory drive leads to a lower steady-state rate for *E*, but this new steady-state value requires a decrease in the *I* firing rate to maintain an appropriate *E*/*I* balance (in effect, the decrease in *E* decreases the drive to *I* by more than the external increase to *I*). This paradoxical effect has profound consequences for plasticity rules that attempt to drive excitatory and inhibitory weights to an activity setpoint.

The relationship of the paradoxical effect and the homeostatic rule performance is presented in Fig. 2*H*. The region of stability for the homeostatic plasticity rules is shown in a parameter regime where inhibitory plasticity is much faster (α*_E_* ≪ α*_I_*). Contrary to when excitatory plasticity dominates, the region of stability is small, and there is no overlap with the region of stability of the neural subsystem. Crucially, the boundary of the stability region of the plasticity rule coincides with the condition for the paradoxical effect to be present (right of the blue line in Fig. 2*H*, *SI Appendix*, Sections 2.2.4 and 2.3.6). Under these conditions, the rules can be stable only when the network is not in an inhibition-stabilized regime. If a network regime with nonzero *E* is forced to exist in that region (e.g., via a tonic external current, Fig. 2*I*), it would be stable only in the nonparadoxical region with the plasticity rules in place (*SI Appendix*, Section 2.5). Note that in the absence of a sufficiently strong external input it is not possible to have stable self-sustained activity that is not inhibition stabilized.

**Fig. 3.**
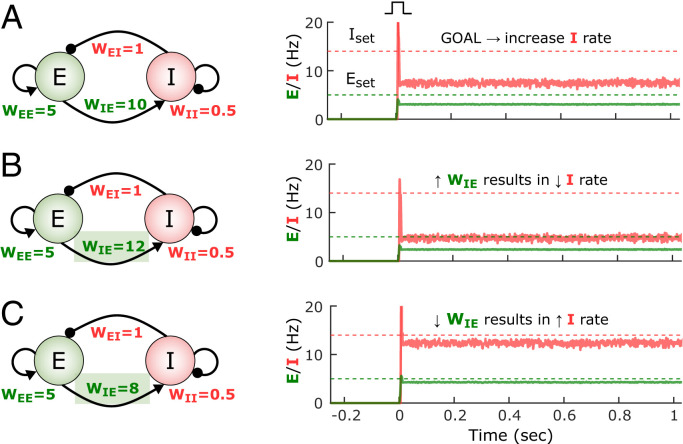
The paradoxical effect constrains the learning rules that can lead to inhibition-stabilized dynamics. (*A*) Example of the self-sustained dynamics of a two-population model in the paradoxical regime with weight values shown in the diagram. Both the *E* and *I* firing rates fall below their respective setpoints. The objective is to adjust the weights so that the *E* and *I* activity match their setpoints. (*B*) An increase of *W_IE_* from 10 to 12 results in a paradoxical decrease of the *I* rate. (*C*) Because of the paradoxical effect an effective way to increase the steady-state *I* firing rate is to decrease its excitatory drive (i.e., *W_IE_*).

To understand the impact of the paradoxical effect on homeostatic plasticity rules, consider a network state in which the *I* rate falls significantly below its setpoint and the *E* rate is close to its setpoint (Fig. 3*A*). In order to reach the *I* setpoint, homeostatic plasticity in the inhibitory neuron would intuitively result in an increase of *W_I__←__E_*. However, because of the paradoxical effect, an increase in *W_I__←__E_* actually makes *I* decrease (Fig. 3*B*), thus increasing the error term *I*_set_ − *I*. To increase the steady-state inhibitory rate, we can “anti-homeostatically” decrease the excitatory weight onto the inhibitory neurons (Fig. 3*C*). (Note that the converse is true for the *W_I__←__I_* weight.) This simple example shows the complexity of designing a coherent set of rules in a strongly coupled system (an analysis of the paradoxical effect is in *SI Appendix*, Section 2.2.4). This analysis also explains why homeostatic plasticity rules can lead to self-sustained activity at the appropriate setpoints when α*_E_* ≫ α*_I_*. Essentially, by allowing plasticity onto the *E* population to be faster, one overcomes the counterproductive homeostatic plasticity associated with the paradoxical effect.

The interaction between the paradoxical effect and homeostatic plasticity in inhibitory neurons leads to the question of whether anti-homeostatic plasticity rules may be more effective that standard homeostatic rules; for example, Δ*W_I__←__E_* ∝ −(*I*_set_ − *I*_avg_). Thus, we also examined a number of hybrid families of plasticity rules with different combinations of homeostatic and anti-homeostatic rules. Indeed, some hybrid families exhibited large degrees of overlap between the stable regions of the network and plasticity rule subsystems. However, numerical simulations revealed that these rules were mostly ineffective in driving networks to self-sustained activity at the target setpoints (*SI Appendix*, Section 1.2 and Fig. S2). These two results are not inconsistent because the stability analysis speaks to cases when the network is initialized to weights that satisfy *E*_set_ and *I*_set_, not whether the rules will drive network activity into these stable areas from any initial state, including an early developmental state. Thus, we interpret these results as meaning that while anti-homeostatic plasticity can contribute to stability of this dual dynamic system, anti-homeostatic plasticity is ineffective at driving the dynamics toward setpoints (in other words, that anti-homeostatic plasticity might allow for stable inhibition-stabilized dynamics but does not necessarily generate sizable basins of attraction around the fixed point).

### Cross-Homeostatic Rule Robustly Leads to the Emergence of Self-Sustained Dynamics.

**Fig. 4.**
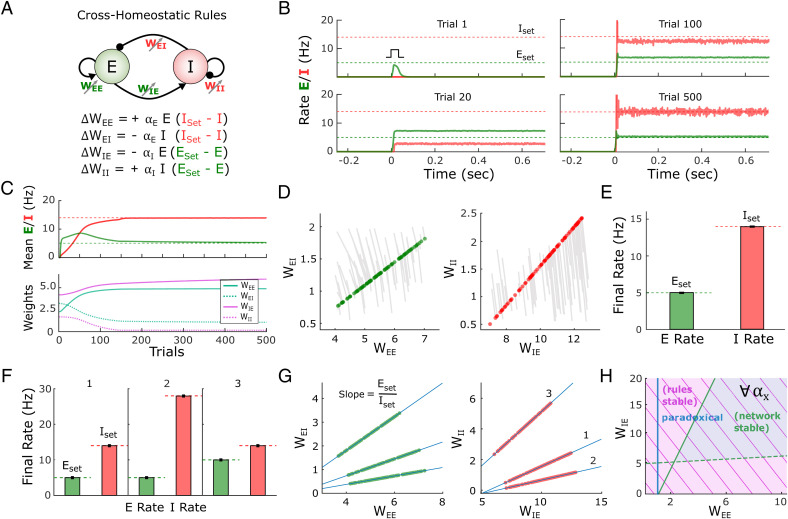
A family of cross-homeostatic rules robustly lead to inhibition-stabilized dynamics at *E*_set_ and *I*_set_. (*A*) Schematic of the network model and the family of cross-homeostatic plasticity rules. (*B*) Example network dynamics across simulated development. The network is initialized with weights that do not lead to self-sustained dynamics in response to an external input (trial 1, weights are initialized to *W_EE_* = 2.1, *W_EI_* = 3, *W_IE_* = 4, and *W_II_* = 2). By trial 20 stable self-sustained activity is observed but at firing rates far from the target setpoints (dashed lines). By trial 500 the network has converged to stable self-sustained activity in which *E* and *I* firing rates match their respective setpoints. The learning rate was set to α*_E_* = α*_I_* = 5*e*^−4^. (*C*) Average rate across trials (*Top*) for the excitatory and inhibitory populations for the data shown in *B*. Weight dynamics (*Bottom*) induced by the cross-homeostatic rules across trials for the data shown in *B*. (*D*) Weight changes for 100 different simulations with random weight initializations (*SI Appendix*, *Supplementary Methods*). Lines show change from initial to final (circles) weight values. (*E*) Average final rates for 100 independent simulations with different weight initializations shown in *D*. Data represent mean ± SEM. (*F*) Final rates for the excitatory and inhibitory subpopulations after plasticity with same starting conditions as in *D* and *E* but for different setpoints. 1: *E*_set_ = 5, *I*_set_ = 14; 2: *E*_set_ = 5, *I*_set_ = 28; 3: *E*_set_ = 10, *I*_set_ = 14. Data shown in *D* and *E* correspond to 1. Data represent mean ± SEM. (*G*) Final weight values for homeostatic plasticity simulations for the three different pairs of setpoints shown in *F*. Blue lines correspond to the theoretical linear relationship between the excitatory and inhibitory weights at a fixed point obeying *E*_set_ and *I*_set_. The slope of the line is defined by the ratio of the setpoints (see *Methods*). (*H*) Analytical stability regions of the neural subsystem and learning rule subsystem as a function of *W_EE_* and *W_IE_*. The stability condition holds for any possible combination of learning rates (*SI Appendix*, Section 1.3).

Given that a standard family of homeostatic plasticity rules did not robustly lead to stable dynamics, we explored alternative learning rules. By defining a loss function based on the sum of the excitatory and inhibitory errors, we analytically derived a set of learning rules using gradient descent (*SI Appendix*, Section 3). This approach led to mathematically complex and biologically implausible rules; however, approximations and simulations inspired a simple class of learning rules that we will refer to as cross-homeostatic (see *Methods*). The main characteristic of this set of rules is that the homeostatic setpoints are “crossed” (Fig. 4*A*). Specifically, the weights onto the excitatory neuron (*W_E__←__E_* and *W_E__←__I_*) are updated to minimize the inhibitory error, while weights into the inhibitory neuron (*W_I__←__E_* and *W_I__←__I_*) change to minimize the excitatory error. Although apparently nonlocal, from the perspective of an excitatory neuron these rules can be interpreted as cells having a setpoint for the total inhibitory input current onto the cell. Such inputs could be read by a cell as the activation of metabotropic receptors (e.g., gamma-aminobutyric acid B [GABA_B_] and metabotropic glutamate; see *Discussion*). Indeed, a similar cross-homeostatic rule has been recently derived for *W_I__←__E_* weights (49).

An example of the performance of the cross-homeostatic rules is shown in Fig. 4 *B* and *C*. After an initial phase with no self-sustained firing (trial 1), recurrent activity reaches stable self-sustained dynamics (trial 20), whose average rate continues to converge toward its defined setpoints (trial 100) until the learning rule system reaches steady state (trial 500). The average *E* and *I* rates of the network evolve asymptotically toward the defined setpoints, as the weights evolve and converge (Fig. 4*C*). Across different weight initializations the rules proved effective in driving the mean activity of the network to the target *E* and *I* setpoints and led to balanced, inhibition-stabilized dynamics (Fig. 4 *D* and *E*). The weight trajectory from its initial value to its final one is shown for 100 different simulations (Fig. 4*D*). Each line corresponds to individual experiments with different initializations (for visualization purposes weights were initialized around the final stable weights; *SI Appendix*, Fig. S3 includes broader initialization conditions). Circles indicate the final values of the weights. Independently of the initial conditions, the weights converge to a line attractor (actually a two-dimensional plane attractor in four-dimensional weight space; *SI Appendix*, Section 2.1). Note that this attractor refers to the sets of weights that generate self-sustained dynamics where *E* and *I* activity matches *E*_set_ and *I*_set_, respectively. That is, for a given pair of setpoints (*E*_set_, *I*_set_) the final values of the weights *W_E__←__I_* and *W_I__←__I_* are linear functions of the “free” weights *W_E__←__E_* and *W_I__←__E_*, respectively. This is a direct consequence of the steady-state conditions for the nontrivial fixed-point of the two-population model (14, 15), where the slope of the line is defined by the setpoints *E*_set_/*I*_set_ (see *Methods*). For example, to satisfy *dE*/*dt* = 0 at the neural activity fixed point, the net excitation and inhibition must obey a specific balance, meaning that once *W_E__←__E_* or *W_E__←__I_* is determined, the other weight is analytically constrained for a given set of setpoints and parameters. Once the weights reach this specific relationship, the *E* and *I* rates reach their corresponding *E*_set_ and *I*_set_ values (Fig. 4*E*). Numerical simulations confirm that the cross-homeostatic rule robustly guides self-sustained activity to different *E*_set_ and *I*_set_ setpoints (Fig. 4*F*), whose ratios define the slopes of the final relationship between the weights (Fig. 4*G*). The above implementations were trial-based, that is, the weights were updated at the end of every trial. An “online” implementation, in which weights were continuously updated, also led to convergence to the setpoints (*SI Appendix*, Fig. S4).

To further validate the effectiveness and stability of the cross-homeostatic rule, we again used analytic methods to determine the eigenvalues of the four-dimensional dynamic system describing the family of four cross-homeostatic rules. As above, stability is determined by the sign of the real part of the eigenvalues of the system. It can be shown (*SI Appendix*, Section 1.3) that these learning rules are stable for any set of parameter values, provided that the stability conditions of the neural subsystem are satisfied (Fig. 4*H*). Importantly, these results demonstrate that cross-homeostatic rules work in both paradoxical and nonparadoxical conditions. Furthermore, the stability of the rules is independent of the absence or presence of external input (*SI Appendix*, Section 2.5). Therefore, it is possible to formally establish that cross-homeostatic learning rules are inherently stable and can robustly account for the emergence and maintenance of self-sustained, inhibition-stabilized dynamics in the two-population model.

### Cross-Homeostatic Rules Drive Average Activity to Setpoints in a Multiunit Model.

**Fig. 5.**
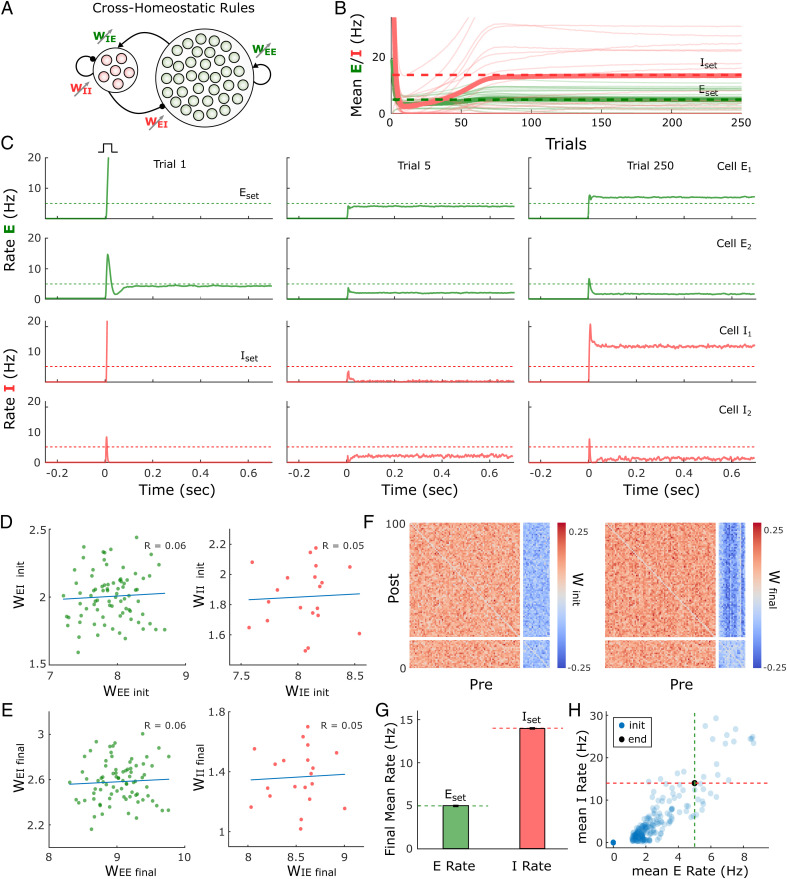
Cross-homeostatic rules drive a multiunit firing rate model to a global network balance. (*A*) Schematic (*Left*) of the multiunit rate model. The network is composed of 80 excitatory and 20 inhibitory units recurrently connected. The four weight classes are governed by cross-homeostatic plasticity rules (*Right*). *SI Appendix*, *Supplementary Methods* includes a detailed explanation of the implementation. (*B*) Evolution of the average rate across trials of 20 excitatory and inhibitory units in an example simulation. The network is initialized with random weights (*SI Appendix*, *Supplementary Methods*), and so neurons present diverse initial rates. *E*_set_ = 5 and *I*_set_ = 14 represent the target homeostatic setpoints. Red and green lines represent the individual (thin lines) and average (thick lines) firing rate of inhibitory and excitatory population, respectively. The learning rate was set to α = 2*e*^−5^. (*C*) Example of the firing rates of two excitatory and two inhibitory units at different points in *B*. The evolution of the firing rates of the excitatory and inhibitory populations within a trial in response to a brief external input is shown in every plot. Individual units converge to stable self-sustained dynamics but not to the defined setpoint. (*D*) *E*–*I* weight relationships at the beginning of the simulation. Every dot represents the total presynaptic weight onto a single unit. *Left,* excitatory neurons; *Right,* inhibitory neurons. (*E*) Same plot as in *D* at the end of the simulation. (*F*) Weight matrix for the multiunit model at the beginning (*Left*) and end (*Right*) of the simulation. Inhibitory weights are shown in blue, excitatory weights in red. (*G*) Average firing rate of the units of the multiunit model and for different initializations of weights (*n* = 400). The network converges to the setpoints in average. Data represent mean ± SEM. (*H*) Same data as in *G* but showing the average initial rate of the network for the multiple initializations (blue dots) and the average rate at the end (black). Target rates are shown in dotted lines (green, *E*_set_ = 5, red *I*_set_ = 14).

The previous results demonstrate the robustness of the cross-homeostatic family of rules in driving a two-subpopulation rate model to a stable self-sustained, inhibition-stabilized regime. We next examined whether these rules are also effective for a multiunit model in which there are many excitatory and inhibitory units. The firing-rate model was composed of 80 excitatory and 20 inhibitory recurrently connected neurons (Fig. 5*A*). In this case, individual neurons adjust their weights to minimize the average error of their presynaptic partners (*SI Appendix*, *Supplementary Methods*). Starting with normally distributed weights, the network reaches stable self-sustained dynamics (Fig. 5 *B* and *C*). However, individual units converge to different final rate values, satisfying the defined setpoints only as an average (green and red thick lines of Fig. 5*B*). This is a result of the nature of the cross-homeostatic rules: Neurons adjust their weights to minimize the error of the mean activity of its presynaptic partners. For this reason, although the network is globally balanced, single units do not converge to the same balanced *E*–*I* line attractor (Fig. 5 *D* and *E*). After cross-homeostatic plasticity, some differential structure is visible among the various weight classes (Fig. 5*F*). Simulations across 400 different initialization conditions demonstrate that the rules lead the average excitatory and inhibitory population activity to *E*_set_ and *I*_set_, respectively (Fig. 5 *G* and *H*). The cross-homeostatic rules are thus capable of driving a multiunit model to a stable self-sustained regime, but they do not guide individual units to local setpoints. Similar results are obtained when the network weights are initialized with log-normal distributions (*SI Appendix*, Fig. S5).

### Learning Rule with Cross-Homeostatic and Homeostatic Terms Leads to Local Convergence to Setpoints.

The above results demonstrate a potential limitation of the cross-homeostatic family of rules: The target setpoints are reached only at the population level. An additional and potentially more serious limitation is that cross-homeostatic rules predict that artificially altering the activity of a small number of excitatory neurons within a large network would not directly produce homeostatic plasticity in these neurons but would directly produce plasticity in their postsynaptic inhibitory neurons. This prediction seems to conflict with homeostatic plasticity experiments that have targeted specific cell types rather than globally altered activity through pharmacological means (50, 51). We therefore assessed the scenario in which both cross-homeostatic and homeostatic rules operate in parallel, resulting in a two-term cross-homeostatic family of rules. Interestingly, this family of rules can be obtained from an approximation of a gradient descent derivation on a loss function that includes the difference between *E* and *I* and their respective setpoints (*SI Appendix*, Section 3). In a two-population model, we first confirmed that this two-term cross-homeostatic family is stable, assuming that the learning rate of the homeostatic term does not dominate (*SI Appendix*, Section 1.4).

**Fig. 6.**
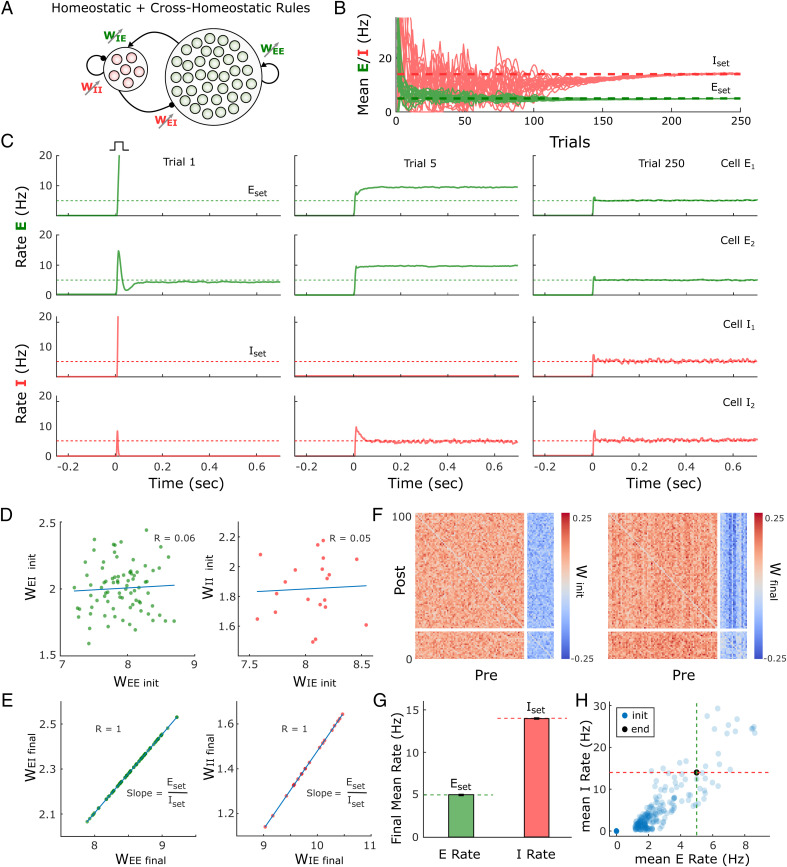
Adding cross-homeostatic influences to homeostatic rules leads to global and local convergence to setpoints. (*A*) Schematic (*Left*) of the multiunit rate model. The network is composed of 80 excitatory and 20 inhibitory units recurrently connected. The four weight classes are governed by homeostatic rules with cross-homeostatic influences (*Right*). *SI Appendix*, *Supplementary Methods* includes a detailed explanation of the implementation. (*B*) Evolution of the average rate across trials in an example simulation (20 excitatory and inhibitory units). The network is initialized with random weights (same as in Fig. 5, *SI Appendix*, *Supplementary Methods*) and so neurons present diverse initial rates. *E*_set_ = 5 and *I*_set_ = 14 Hz represent the target homeostatic setpoints. The learning rate was set to α = 1*e*^−5^. (*C*) Example of the firing rate of two excitatory and two inhibitory units at different points in *B*. The evolution of the firing rate of the excitatory and inhibitory population within a trial in response to a brief external input is shown in every plot. Units converge to stable self-sustained activity and at an individual setpoint. (*D*) *E*–*I* weight relationships at the beginning of the simulation. Every dot represents the total presynaptic weight onto a single unit. *Left,* excitatory neurons; *Right,* inhibitory neurons. (*E*) Same plot as in *D* at the end of the simulation. The network has reached a stable state and weights converge to single *E*–*I* balance defined by a line attractor. (*F*) Weight matrix at the beginning (*Left*) and end (*Right*) of the simulation. Inhibitory weights are shown in blue, excitatory weights in red. (*G*) Average firing rate of the units of the multiunit model and for different initializations of weights (*n* = 400). Data represent mean ± SEM. (*H*) Same data as in *G* but showing the average initial rate of the network for the multiple initializations (blue dots) and the average rate at the end (overlapping black circles). Target rates are shown in dotted lines (green, *E*_set_; red, *I*_set_).

Simulations with the same multiunit model as in Fig. 5 show that with the two-term cross-homeostatic rule all individual units converge to their respective *E*_set_ and *I*_set_ (Fig. 6 *A*–*C*). Importantly, in contrast to the single-term cross-homeostatic rule, the total excitatory and inhibitory weight of each individual unit converged to the *E*–*I* balance of the line attractor predicted by the network equations (Fig. 6 *D* and *E*), while some structure in the different weight classes is also observed in the connectivity matrices (Fig. 6*F*). The convergence to the setpoints was stable across a wide range of initial states (Fig. 6 *G* and *H*). Thus, a hybrid family of plasticity rules that includes both cross-homeostatic and homeostatic forces provides global network stability while also locally driving each unit to their setpoint and a balanced *E*–*I* regime.

### Spiking Neural Network Model with Sparse Connectivity Converges to an Inhibition-Stabilized Regime at the Setpoints.

**Fig. 7.**
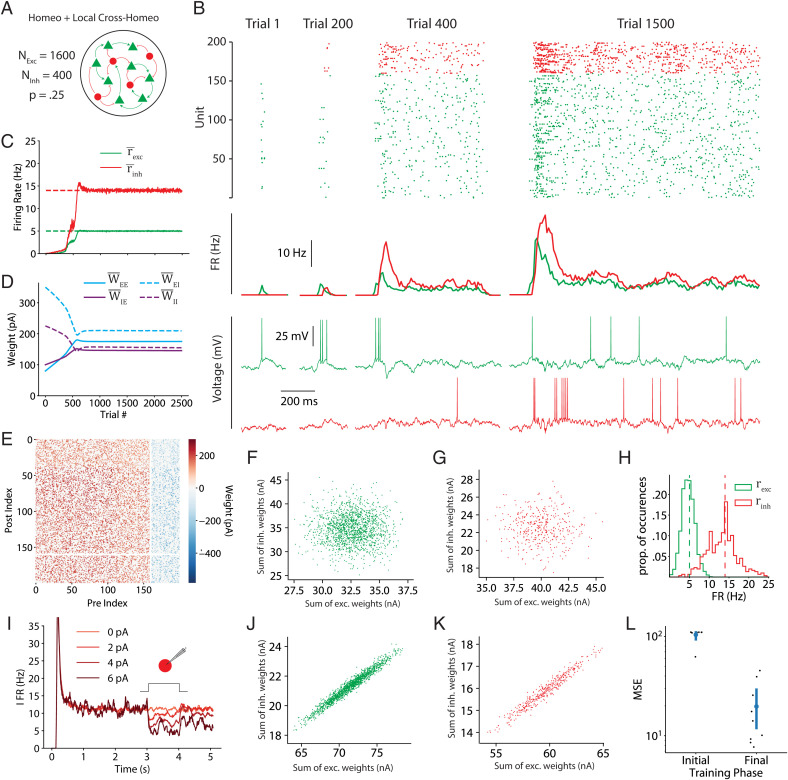
Two-term cross-homeostatic learning rules guide a sparse spiking network model to an inhibition-stabilized regime at the target setpoints. (*A*) Schematic of the network. Two thousand leaky adaptive integrate-and-fire units (1,600 excitatory and 400 inhibitory) were connected with a 25% probability. (*B*) Spike rasters, population PSTHs (peristimulus time histograms), and sample voltage traces for excitatory (green) and inhibitory (red) units, across four stages over the course of training. (*C*) Population average firing rates over the course of training (moving average with a width of five trials). (*D*) Mean weights for each synaptic class over the course of training. Weights were initialized in an early developmental regime reflecting a silent network. $\bar{W_{EE}}=80\;pA,\;\bar{W_{IE}}=100\;pA,\;\bar{W_{EI}}=350\;pA,\;\bar{W_{II}}=225\;pA$. (*E*) Weight matrix at the end of training. Due to the size of the network, only a 10% subset of the full weight matrix is shown. (*F*) Initial *E*/*I* balance onto excitatory units, visualized as a scatterplot of the sum of incoming excitatory synaptic weights versus the sum of incoming inhibitory weights onto each excitatory unit. (*G*) Same as *F* but for the initial *E*/*I* balance onto inhibitory units. (*H*) Histograms of the firing ratess for each excitatory and inhibitory population at the end of training (dashed lines represent the setpoints). (*I*) At the end of training, the network exhibits the paradoxical effect. When a positive current is injected into all inhibitory units, the mean inhibitory firing rate decreases. This effect is visualized with PSTHs of the inhibitory population firing rate across 40 trials at each of the injected current values. (*J*) Final *E*/*I* balance onto excitatory units, visualized as a scatterplot of the sum of incoming excitatory synaptic weights versus the sum of incoming inhibitory weights onto each excitatory unit. (*K*) Same as *J* but for the final *E*/*I* balance onto inhibitory units. (*L*) Robustness of convergence to weight initialization for nine networks initialized with different mean weights, we show the initial and final MSE of the unit FRs with respect to their homeostatic setpoints after a 6,000-trial training session.

The previous results demonstrate the ability of the two-term cross-homeostatic plasticity rule to guide firing-rate-based models to inhibition-stabilized regimes at the target setpoints. We next examined the effectiveness of this family of learning rules in a sparsely connected spiking neural network (Fig. 7*A*). In a sparsely connected network, the cross-homeostatic component of the learning rule can be implemented globally (e.g., excitatory plasticity onto an excitatory unit is based on the mean error of the entire population of inhibitory units) or locally (e.g., excitatory plasticity onto an excitatory neuron is based on the mean error of its presynaptic inhibitory partners). Here we used a local implementation, in which, for example, an excitatory neuron has a setpoint interpreted as a target for the total amount of GABA_B_ receptor activation it should receive (see *Discussion*).

Starting from a developmental scenario with weak weights (i.e., that do not support any self-sustaining activity), the rules successfully drive the network to stable self-sustained and asynchronous spiking activity near the setpoints (Fig. 7 *B*–*E*; CV_ISI_∼1). As seen in the firing rate multiunit model, the weights self-organize from an unstructured initial condition (Fig. 7 *F* and *G*) to a state where a balance of excitation and inhibition emerges (Fig. 7 *H–K*). After training, the firing rates of the excitatory and inhibitory neurons distribute around their setpoints (Fig. 7*H*). As expected, the emergent network dynamics exhibits the paradoxical effect; specifically, when inhibitory neurons are transiently activated with an external current, a net decrease in the mean firing rate is observed (Fig. 7*I*). Finally, convergence holds across networks initialized with different weights (Fig. 7*L*). These results demonstrate that the theoretical and computational results in rate networks translate to more complex and biologically realistic scenarios.

## Discussion

Elucidating the learning rules that govern the connectivity within neural circuits is a fundamental goal in neuroscience, in part because learning rules establish unifying principles that span molecular, cellular, systems, and computational levels of analyses. Elucidation of Hebbian associative synaptic learning, for example, linked simple computations at the level of single proteins (the *N*-methyl-d-aspartate receptor) with higher-order computations at the system and computational levels (52–56). However, it remains the case that because most studies have focused on learning rules at one or two synapse classes, little is known about the learning rules that give rise to complex neural dynamic regimes. Here we have taken steps toward exploring families of learning rules that operate in parallel at four different synapse classes, and starting from a silent state, capture the experimentally observed emergence of self-sustained, inhibition-stabilized dynamics in cortical networks.

We first explored whether standard formulations of homeostatic plasticity can account for the unsupervised emergence of self-sustained, inhibition-stabilized regimes. Based on experimental data we assumed that both excitatory and inhibitory neurons have an ontogenetically programmed activity setpoint during self-sustained activity and that plasticity in the four weight classes is driven by standard formulations of homeostatic plasticity. Numerical simulations and analytical stability analyses revealed that while some initial conditions and parameter regimes led to self-sustained dynamics, they occupied a narrow region of parameter space, when the rate of synaptic plasticity onto inhibitory neurons is much lower than that onto excitatory neurons (Fig. 2*G* and *SI Appendix*). When the rates of inhibitory and excitatory plasticity are comparable, analytical stability analyses confirmed that the region of stability of the network dynamics overlapped only in a narrow region. Such a narrow stability area seems incompatible with the robustness necessary in biological systems and with experimental data showing that inhibitory neurons exhibit homeostatic plasticity as fast as or faster than excitatory neurons (40–42, 47, 48). We thus conclude that a family of standard homeostatic plasticity rules operating in all four synapse classes is not sufficient to account for the experimentally observed emergence of self-sustained dynamics in cortical circuits.

### Cross-Homeostatic Plasticity.

Analyses of approximations of a gradient-descent-derived learning rule suggested, somewhat counterintuitively, that adjusting the *E* population based on the error of the *I* population (and vice versa) may prove to be an effective family of learning rules. Indeed, numerical simulations and analytical stability analyses revealed that this cross-homeostatic rule was robustly stable (Fig. 4). However, the convergence to the excitatory and inhibitory setpoints in a multiunit network occurred only at the population level, not at the level of individual units. This observation is not inconsistent with experimental data, which show that in vivo neurons do exhibit a wide range of variability in their apparent setpoints (57, 58). However, a significant concern with this single-term cross-homeostatic rule is that it predicts that selectively increasing activity in a subpopulation of excitatory neurons would first induce plasticity in inhibitory neurons (*W_I_*_←_*_E_* and *W_I_*_←_*_I_*), which could in turn lead to plasticity in the manipulated excitatory neurons (*W_E_*_←_*_E_* and *W_E_*_←_*_I_*). Most homeostatic plasticity studies do not speak to this prediction because they have used pharmacological manipulations of both excitatory and inhibitory neurons. However, some studies have used cell-specific manipulations—such as cell-specific overexpression of potassium channels (50, 51)—that strongly support the notion that synaptic plasticity is guided at least in part by their own deviation from setpoint.

In our opinion, and although we have explored alternative rules (*SI Appendix*, Section 1.6), the most biologically plausible set of plasticity rules that lead to stable self-sustained dynamics comprises a hybrid rule that includes both standard homeostatic and cross-homeostatic terms. Such a two-term cross-homeostatic rule robustly led to a self-sustained, inhibition-stabilized network, with all units converging to their setpoints, and is directly consistent with current experimental data.

### Biological Plausibility of Cross-Homeostatic Plasticity.

While the neural mechanisms underlying homeostatic plasticity remain to be elucidated, it is generally assumed that an individual neuron can maintain a running average of their firing rate over the course of hours as a result of Ca^2+^-activated sensors. Based on the deviation of this value from an ontogenetically determined setpoint, neurons up- or down-regulate the density of postsynaptic receptors accordingly (38, 58–60). Two-term cross-homeostatic plasticity would require additional, and apparently nonlocal information about the error in a given neuron’s presynaptic partners. Importantly however, this rule can be implemented locally because any postsynaptic neuron has access to the mean activity of its presynaptic partners simply as a result of its postsynaptic receptor activation. Indeed, a plasticity rule for *W_I__←__E_* weights with a similar cross-homeostatic error term has also been recently proposed and implemented based on the mean activation of postsynaptic receptors—more specifically the net postsynaptic currents, which provide a coupled measure of average presynaptic firing and synaptic weights (49).

Here we propose that cross-homeostatic plasticity could be implemented through postsynaptic metabotropic receptors (e.g., mGlu and GABA_B_). Such receptors would provide a mechanism for postsynaptic neurons to maintain a running average of the activity of its presynaptic partners that is decoupled from the synaptic weights. Metabotropic receptors are G protein coupled receptors that provide a low-pass filtered measure of presynaptic activity and are involved in a large number of incompletely understood neuromodulatory roles (61, 62). Since metabotropic receptors appear to undergo less homeostatic and associative plasticity, they provide a measure of presynaptic activity that is naturally decoupled from the ionotropic receptors (e.g., AMPA and GABA_A_) that are being up- and down-regulated.

Further support for the notion that individual neurons have access to global network activity emerges from studies suggesting that neurons might not homeostatically regulate activity at the individual neuron level but rather at the global population level (63). Such a global-level homeostasis could be achieved by nonsynaptic paracrine transmission. Indeed, retrograde messenger systems are ideally suited for this role, as they have already been implicated in signaling mean activity levels to local capillaries, driving the activity-dependent vasodilation that underlies functional MRI (64).

### Paradoxical Effect and Standard Homeostatic Rules.

The paradoxical effect is one of the defining features of inhibition-stabilized networks, and a growing body of evidence suggests that the cortex operates in this particular dynamic regime (20, 65–67). As recent work has begun to hint (68), here we formally prove that the paradoxical effect applies important constraints to the potential learning rules that lead to the emergence of inhibition-stabilized networks. In the simplified case in which there is only homeostatic plasticity onto the inhibitory neurons, we can immediately see why the paradoxical effect renders standard homeostatic rules ineffective. If the *I* population is below its setpoint, standard homeostatic rules would increase *W_I__←__E_*, which paradoxically would further decrease *I* (Fig. 3), thus further increasing the error instead of decreasing it (Fig. 3). This reasoning is related to why, when using the standard family of homeostatic rules, the rate of plasticity onto the inhibitory neurons has to be much smaller—in effect making the “paradoxical homeostatic plasticity effect” much slower. Furthermore, our analytical stability analyses show that in the limit of vanishingly small excitatory learning rates (α_EE,EI_ ≪ α_IE,II_) the stability region of the weight subsystem is bounded by the paradoxical condition. This means that the only allowed stable states with nonzero *E* activity will occur in the nonparadoxical regime, if any, and they will not be proper inhibition-stabilized, self-sustained regimes.

### Future Directions and Experimental Predictions.

While we have taken the approach of implementing homeostatic plasticity rules at all four synapse classes in our model, it is important to stress that we have omitted other well-characterized forms of synaptic plasticity. In particular, we did not include associative long-term potentiation or spike-timing dependent plasticity. These forms of plasticity are generally considered to capture the correlation structure in networks that are driven by structured inputs. Arguably, because there is evidence that self-sustained forms of activity such as up-states develop in the absence of any structured external input (23, 25, 26) and because all excitatory and inhibitory neurons synchronously shift between quiescent and active states, associative forms of plasticity may not contribute significantly to these regimes. Nevertheless, we envision the cross-homeostatic rules we propose working hand in hand with associative forms of plasticity that impose high-dimensional structure on the top of the inhibition-stabilized dynamics. In fact, preliminary observations reveal that cross-homeostatic rules are capable of stabilizing recurrent networks while preserving imposed Hebbian-like structure in the weight matrix (*SI Appendix*, Fig. S6). Future work should explore the computational advantage of cross-homeostatic plasticity in models performing complex computational tasks, such as working memory, sensory timing, or motor control (5, 69–72).

An important implication of our results is that neuronal and network properties can operate in fundamentally different modes. That is, while homeostatic plasticity can lead to single neurons to reach their target setpoints in simple feedforward circuits, those same rules can be highly unstable when the neurons are placed even in the simplest of recurrent excitatory/inhibitory circuits with emergent dynamics. Furthermore, because emergent neural dynamic regimes are highly nonlinear, and in particular that stable self-sustained dynamic regimes exhibit a paradoxical effect, it is likely that the brain exhibits paradoxical or counterintuitive learning rules to generate self-sustained dynamic regimes.

## Methods

### Computational Model.

A two-population firing-rate model was implemented based on a previous inhibition-stabilized network model (19). The firing rate of the excitatory (*E*) and inhibitory (*I*) population obeyed Wilson and Cowan dynamics (73).

[1] $$\tau_{E}\frac{dE}{dt}=-E\left(t\right)+f_{E}\left(W_{EE}E\left(t\right)-W_{EI}I\left(t\right)+\eta_{E}\left(t\right)\right),$$

[2] $$\tau_{I}\frac{dI}{dt}=-I\left(t\right)+f_{I}\left(W_{IE}E\left(t\right)-W_{II}I\left(t\right)+\eta_{I}\left(t\right)\right),$$

where *W_XY_* represents the weight between the presynaptic unit *Y* and postsynaptic unit *X*. The parameters τ*_X_* and η*_X_* represent a time constant and an independent noise term, respectively. The time constants were set to τ*_E_* = 10 ms for the excitatory and τ*_I_* = 2 ms for the inhibitory subpopulations. The noise term was an Ornstein–Uhlenbeck process with mean μ*_x_* = 0, a time constant 1/Θ*_x_* = 1 ms, and a sigma parameter of σ*_x_* = 10. To elicit self-sustained activity, a step current was injected at the beginning of each trial on the excitatory population.

The function *f_Y_*(*x*) represents the intrinsic excitability of the neurons, and it is modeled as a threshold-linear function with threshold θ*_Y_* and gain *g_Y_*.

[3] $$f_{Y}\left(x\right)=\{\begin{array}{c} 0\;if\;x<\theta_{Y}\\ g_{Y}\left(x-\theta_{Y}\right)if\;x\geq \theta_{Y} \end{array},\;Y=\{E,I\}.$$

The thresholds were set to θ*_E_* = 4.8 and θ*_I_* = 25, and the gains to *g_E_* = 1 and *g_I_* = 4. The higher thresholds in PV neurons are consistent with experimental findings (46).

The linear relationship between excitatory and inhibitory weights (Fig. 4) corresponds to the steady-state solution of the neural subsystem when the inhibitory and excitatory rates are at their target setpoints. The solution can be obtained by setting the left side of Eqs. **1** and **2** to zero and substituting the steady-state E and I values with *E*_set_ and *I*_set_:

[4] $$W_{EI}=\frac{W_{EE}E_{\mathrm{Set}}}{I_{\mathrm{Set}}}-\frac{\theta_{E}g_{E}+E_{\mathrm{Set}}}{I_{\mathrm{Set}}g_{E}},$$

[5] $$W_{II}=\frac{W_{IE}E_{\mathrm{Set}}}{I_{\mathrm{Set}}}-\frac{\theta_{I}g_{I}+I_{\mathrm{Set}}}{I_{\mathrm{Set}}g_{I}}.$$

Thus, the slope of the *E*/*I* balance line in Fig. 4 corresponds to *E*_set_/*I*_set_. We chose *W_E←E_* and *W_I←E_* as the “free” weights. See details and analytical results in *SI Appendix*, Section 2.2.

### Synaptic Plasticity.

Plasticity at all four weight classes (*W_E←E_*, *W_E←I_*, *W_I←E_*, and *W_I←I_*) was governed by different families of homeostatic-based plasticity rules, all driven by the deviation of the actual excitatory and inhibitory rates from their target setpoints (*E*_set_ and *I*_set_). Three different learning rules are presented in the main text of this article.

#### Standard homeostatic family of rules.


[6] $$\begin{array}{l} \Delta W_{EE}=+\alpha_{E}E\left(E_{\mathrm{set}}-E\right)\\ \Delta W_{EI}=-\alpha_{E}I\left(E_{\mathrm{set}}-E\right)\\ \Delta W_{IE}=+\alpha_{I}E\left(I_{\mathrm{set}}-I\right)\\ \Delta W_{II}=-\alpha_{I}I\left(I_{\mathrm{set}}-I\right), \end{array}$$

where α*_E_* and α*_I_* are the learning rates onto the excitatory and inhibitory units, respectively. The setpoints were based on empirically measured values in ex vivo cortical circuits (46), *E*_set_ = 5 and *I*_set_ = 14 Hz and follow a classic homeostatic formulation (33, 34, 43, 44). As outlined in *SI Appendix*, Section 1.5, we also examined variants of these rules, such as standard synaptic scaling (which includes the weight as a factor).

We prove that these rules are stable only in a narrow parameter regime: when excitatory plasticity dominates (*SI Appendix*, Section 2).

#### Cross-homeostatic family of rules.


[7] $$\begin{array}{l} \Delta W_{EE}=+\alpha_{E}E\left(I_{\mathrm{set}}-I\right)\\ \Delta W_{EI}=-\alpha_{E}I\left(I_{\mathrm{set}}-I\right)\\ \Delta W_{IE}=-\alpha_{I}E\left(E_{\mathrm{set}}-E\right)\\ \Delta W_{II}=+\alpha_{I}I\left(E_{\mathrm{set}}-E\right). \end{array}$$

These rules differ from the standard homeostatic formulation in that the setpoints are “crossed,” meaning that the weights onto the excitatory (inhibitory) population change in order to minimize the inhibitory (excitatory) error. We prove that these rules are stable for any set of parameters (*SI Appendix*, Section 1.3).

#### Two-term cross-homeostatic family of rules.


[8] $$\begin{array}{l} \Delta W_{EE}=+\alpha_{E}E\left(E_{\mathrm{set}}-E\right)+\alpha_{E}E\left(I_{\mathrm{set}}-I\right)\\ \Delta W_{EI}=-\alpha_{E}I\left(E_{\mathrm{set}}-E\right)-\alpha_{E}I\left(I_{\mathrm{set}}-I\right)\\ \Delta W_{IE}=+\alpha_{I}E\left(I_{\mathrm{set}}-I\right)-\alpha_{I}E\left(E_{\mathrm{set}}-E\right)\\ \Delta W_{II}=-\alpha_{I}I\left(I_{\mathrm{set}}-I\right)+\alpha_{I}I\left(E_{\mathrm{set}}-E\right). \end{array}$$

The two-term rules combine homeostatic and cross-homeostatic terms. This exact formulation can be obtained after an approximation of a gradient descent derivation on the following loss function:

[9] $$L=\frac{1}{2}{\left(E-E_{\mathrm{set}}\right)}^{2}+\frac{1}{2}{\left(I-I_{\mathrm{set}}\right)}^{2}.$$

The mathematical derivation can be found in the *SI Appendix*, Section 3. We prove that these rules are stable for a biologically meaningful set of parameter values, as long as the homeostatic part does not dominate (*SI Appendix*, Section 1.4).

For all other methods, including the implementation of the multiunit firing rate and spiking models, numerical and analytical methods, proofs, and derivation of the two-term cross-homeostatic rule, see the *SI Appendix*.

## Supplementary Material

Supplementary File

## Data Availability

Software necessary to reproduce all data shown throughout this manuscript is fully available in the public repository GitHub (see links below). Computational analysis for the rate-based model was performed in custom-written MATLAB R2020a software (https://www.mathworks.com/). Brian2 was used to simulate the spiking model (https://briansimulator.org/). SageMath was used for the analytical proofs (https://www.sagemath.org/) (*SI Appendix*). The MATLAB source code that reproduces Figs. 2, 4–6 and *SI Appendix*, Fig. S4 is available at https://github.com/saraysoldado/Paradoxical2022 (74). The spiking model can be found at https://github.com/mikejseay/spiking-upstates/tree/Paradoxical2022 (75). The Jupyter notebooks with SageMath code to reproduce all analytical results are available at https://github.com/SMDynamicsLab/Paradoxical2022 (76).
