# Supplementary material for: Paradoxical self-sustained dynamics emerge from orchestrated excitatory and inhibitory homeostatic plasticity rules
Source: Proc Natl Acad Sci U S A. 2022 Oct 17;119(43):e2200621119. doi: 10.1073/pnas.2200621119 (PMC9618084; doi:10.1073/pnas.2200621119)
Supplement: Supplementary File [file pnas.2200621119.sapp.pdf]

# Supplementary Information for

## Paradoxical Self-Sustained Dynamics Emerge from Orchestrated Excitatory and Inhibitory Homeostatic Plasticity Rules

Saray Soldado-Magraner, Michael J. Seay, Rodrigo Laje, Dean V. Buonomano

**This PDF file includes:**

-Supplementary Figures

- Supplementary Figures S1 to S7

-Supplementary Methods

-Supplementary Material (Analytical results and derivations)

## Supplementary Figures

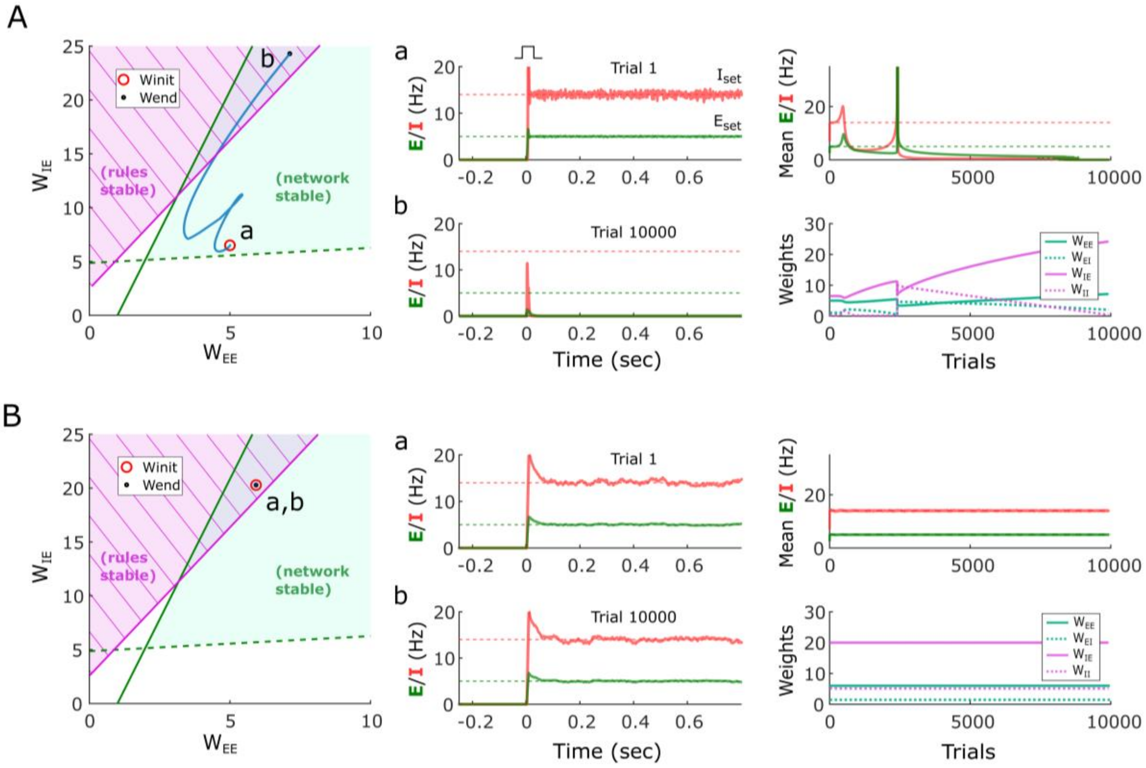


**Figure S1. Stability is achieved only in the region where both the neural and synaptic plasticity rule subsystems are stable.**

**(A)** Example of a network initialized within the stability region of the neural subsystem, but within the unstable region of the plasticity rule subsystem. At the beginning of the simulation the network is in a stable self-sustained activity (a). Specifically, in addition to the "free" $W_{EE}$ and $W_{IE}$ weights shown in the plot, the $W_{EI}$ and $W_{II}$ weights are set according to the steady state solution for activity fixed point (loosely speaking $W_{EE}/W_{EI}$ and $W_{IE}/W_{II}$ are "balanced", see Eqs. 4 and 5). Under the standard homeostatic rules, the weights of the network diverge (blue trajectory), and with time the stable self-sustained activity is no longer observed (b). Right panels show the evolution of the average firing rate and weights across trials. The firing rate diverges under the presence of the rules, and explodes (until reaching saturation, see Methods), finally settling in a Down-state. The weights keep evolving unbounded. The initial weights are $W_{EE}=5$ and $W_{EI}=6.5$ ($W_{EI}$ and $W_{II}$ are set according to Eqs. 4 and 5, see Methods). Note that during plasticity the $W_{EE}$ and $W_{IE}$ weights may cross the overlapping region in which the plasticity rule (hatched) and network (green) subsystems are stable, but still not converge because the $W_{EI}$ and $W_{II}$ weights have also been evolving and are no longer "balanced" with $W_{EE}$ and $W_{IE}$, respectively.

**(B)** Example of a network initialized within the stability region of the neural subsystem, and within the stable region of the plasticity rule subsystem. At the beginning of the simulation the network is on a stable self-sustained activity (a). In this case, despite the presence of the homeostatic rules, the weights of the network do not diverge and the network remains in a stable self-sustained regime (b). Right panels show the evolution of the average firing rate and weights across trials. The firing rate and weights remain stable across trials. The initial weights are $W_{EE}=6$ and $W_{EI}=20$ ($W_{EI}$ and $W_{II}$ are set according to equations 4 and 5, see Methods).

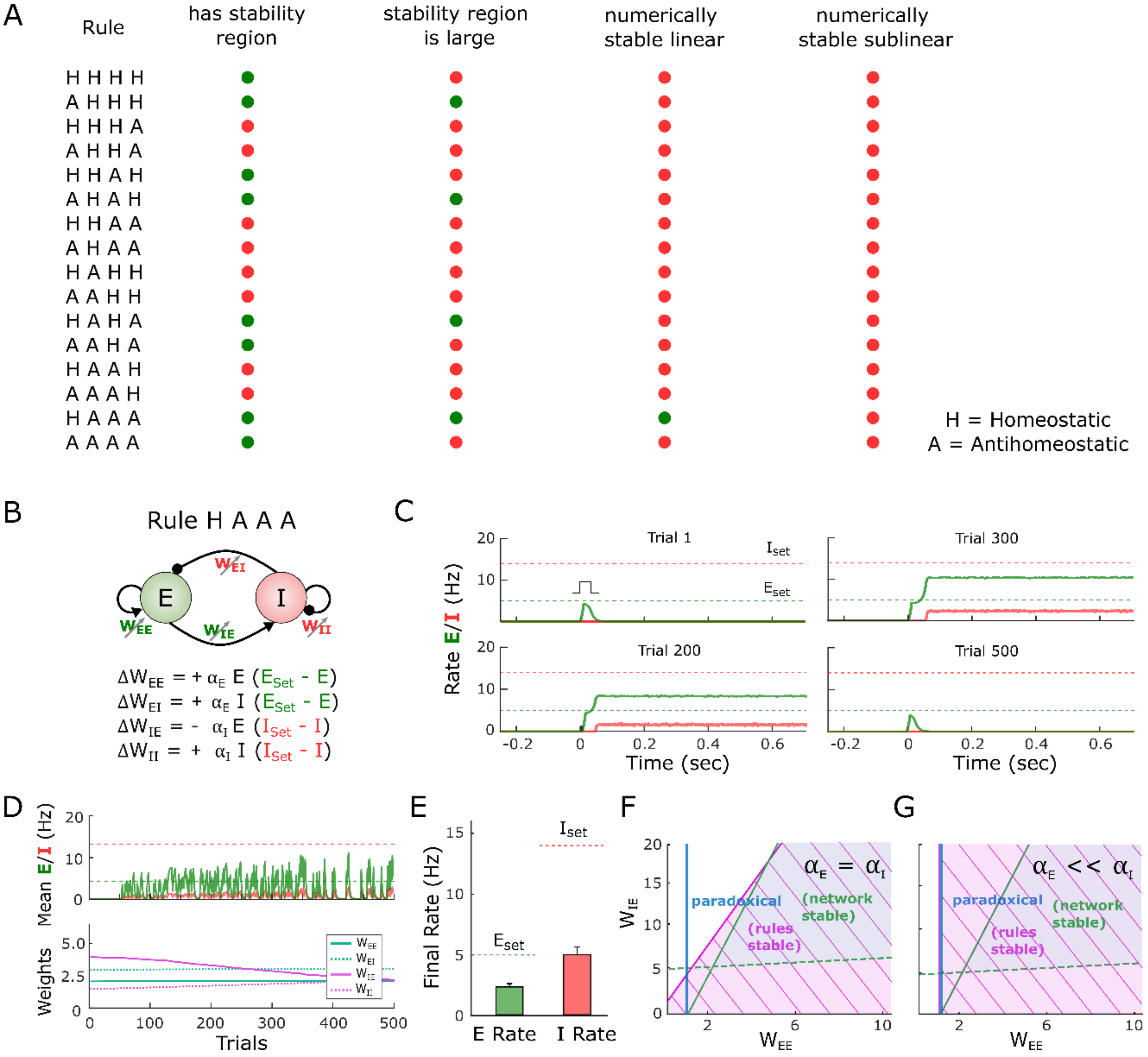


**Figure S2. Homeostatic and anti-homeostatic combinations of plasticity rules also fail to drive the emergence of self-sustained dynamics.**

**(A)** Sixteen variations of the standard homeostatic rules presented in **Fig. 2** were assessed for stability. The plasticity governing each of the four weight types, $W_{EE}$, $W_{EI}$, $W_{IE}$, $W_{II}$ was set to be either homeostatic (H) or antihomeostatic (A). The first rule on the table (HHHH) corresponds to the standard homeostatic rules presented in **Fig. 2**, where all weights obey homeostatic plasticity. All rules were tested for stability analytically and numerically. A red dot implies that the listed condition is not satisfied, while a green dot means that it does. The condition on the first column indicates whether a stability region for the plasticity rule is present. The second column indicates whether such region has a large overlap with the region of stability of the neural subsystem. The third column indicates whether the rule is successful, using numerical simulations, at driving the network to a stable self-sustained activity when starting from regimes with self-sustained activity already present (meaning the network is initialized in the linear regime). The fourth column indicates the same as the former, but with the network initialized in the sub-linear regime, where activity is not initially present (e.g., as observed early in developmental conditions).

**(B)** Schematic (top) of the population rate model in which the four weights are governed by the HAAA rule in panel (A).

**(C)** Example simulation of the HAAA rule over the course of simulated development. The evolution of the firing rate of the excitatory and inhibitory population within a trial in response to a brief external input is shown in every plot. $E_{set}=5$ and $I_{set}=14$ represent the target homeostatic setpoints. Weights were initialized to $W_{EE}=2.1$, $W_{EI}=3$, $W_{IE}=4$, and $W_{II}=2$ as in **Fig. 2**. Note that while an evoked self-sustained activity emerges by Trial 200 the firing rates do not converge to their setpoints, and by Trial 500 the stable activity is no longer observed.

**(D)** Average rate across trials (upper plot) for the excitatory and inhibitory populations for the data shown in **C**. Weight dynamics (bottom plot) produced by the homeostatic rules across trials for the data shown in **C**.

**(E)** Average final rate for 100 independent HAAA simulations with different weight initializations. Those initializations included cases in which the network starts in the sublinear regime (where the initial *E* firing rate was zero or very low). The weights were initialized uniformly between the following ranges: $W_{EE}$[1,3], $W_{EI}$[0.5,1.5], $W_{IE}$[4,8], $W_{II}$[0.2,0.8]. Data represents mean ± SEM.

**(F)** Analytical stability regions of the neural and HAAA plasticity rule subsystems as a function of the free weights $W_{EE}$ and $W_{IE}$. Here the stability plot is obtained by considering equal learning rates for all four plasticity rules (as used for panels **C-E**).

**(G)** Similar to **F** but with but with $\alpha_E << \alpha_I$. Right of blue line shows the area where the network is in a paradoxical regime (defined by the condition $W_{EE} * g_E - 1 > 0$). Contrary to standard homeostatic rules (**Fig. 2**), the HAAA rule is only stable in the paradoxical region of parameter space (i.e., $W_{EE} * g_E - 1 > 0$; note white area to the left of the blue line). This may explain why the rule fails at driving the network to a stable self-sustained activity when starting with developmental-like conditions.

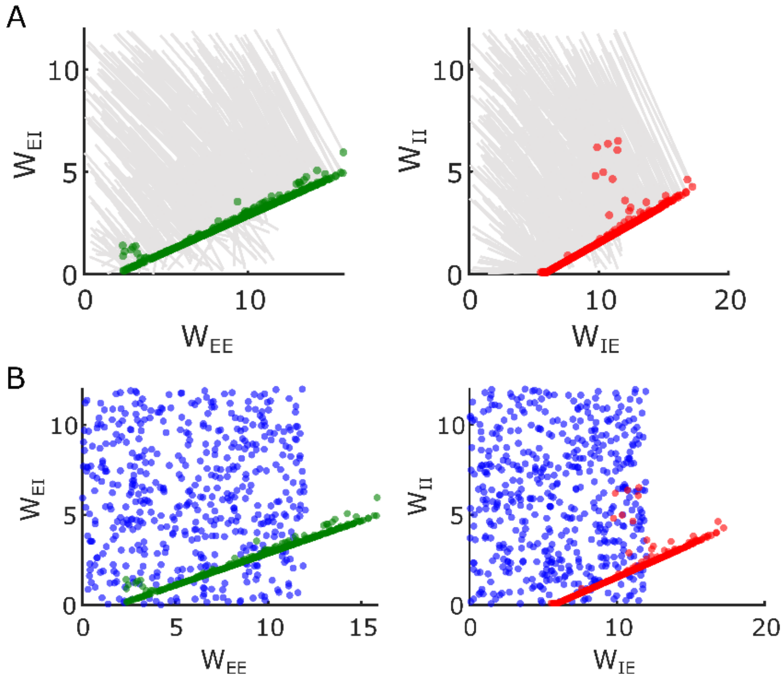


**Figure S3. A broader weight initialization also leads to converge of the cross-homeostatic rules in the two-population model.**

**(A)** Same as in **Fig.4D** weight changes for 100 different simulations with random weight initializations are shown. Lines show change from initial to final (circles) weight values. Weights are initialized uniformly between the following ranges: $W_{EE}$[0,12], $W_{EI}$[0,12], $W_{IE}$[0,12], $W_{II}$[0,12].

**(B)** Same data as in (A) but only displaying the initial weight values (blue circles) and the final ones (green and red circles).

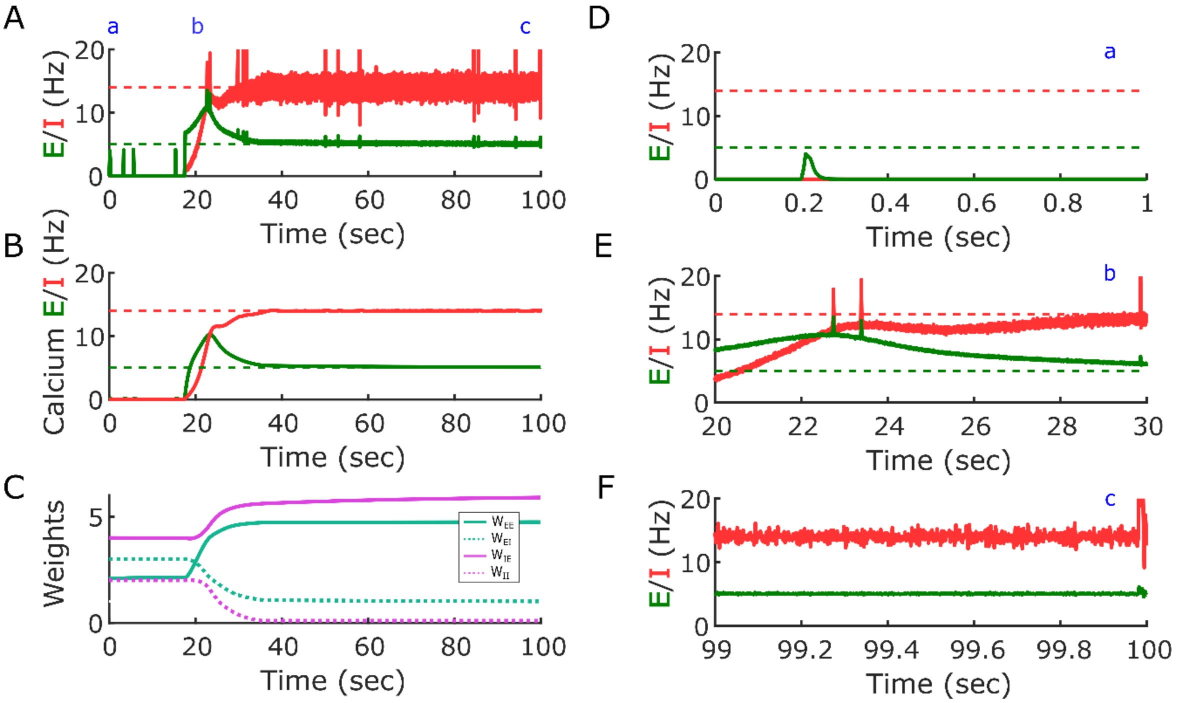


**Figure S4. An online implementation of cross-homeostatic plasticity in the two-population model also converges to inhibition-stabilized dynamics at the setpoints.**

**(A)** Firing rate of the excitatory and inhibitory population over time. Cross-homeostatic plasticity implemented in an online or continuous fashion (as opposed to trial-based updates of the weights) also drives the network from a silent state to its setpoints at $E_{set}$=*5* and $I_{set}$=14 (dashed lines). Random Poisson input arrives at a frequency of 0.1 Hz to engage recurrent activity ($I_{extt}$=7, $I_{dur}$=10 ms).

**(B)** A calcium sensor in both, the excitatory and inhibitory population continuously integrates their activity with $\tau_{Ca^{2+}} = 1000\, ms$ .

**(C)** The weights are updated at every time step based on the instantaneous calcium sensor value. Weights are initialized as in **Fig.4** $W_{EE}$=2.1, $W_{EI}$=3, $W_{IE}$=4, and $W_{II}$=2. Learning rate is set to $\alpha = 5e^{-7}$. The rest of the network parameters are the same as in **Fig.4.**

**(D)** Snippet of the first second of simulation time shown in **A.** This time point is shown as a blue a). The network starts in a silent state as in **Fig.4B.** The first external input to the network is set manually for comparison. The rest of the inputs arrive at random Poisson times with a frequency of 0.1 Hz.

**(E)** Snippet of the activity of the network between seconds 20 and 30 of simulation time (b). The firing rate of the excitatory and inhibitory connectivity raises towards its setpoints. Note the Poisson external input is still present but it does not disturb the network stability.

**(F)** Last second of simulation time (c). The activity of the network has converged to its setpoints.

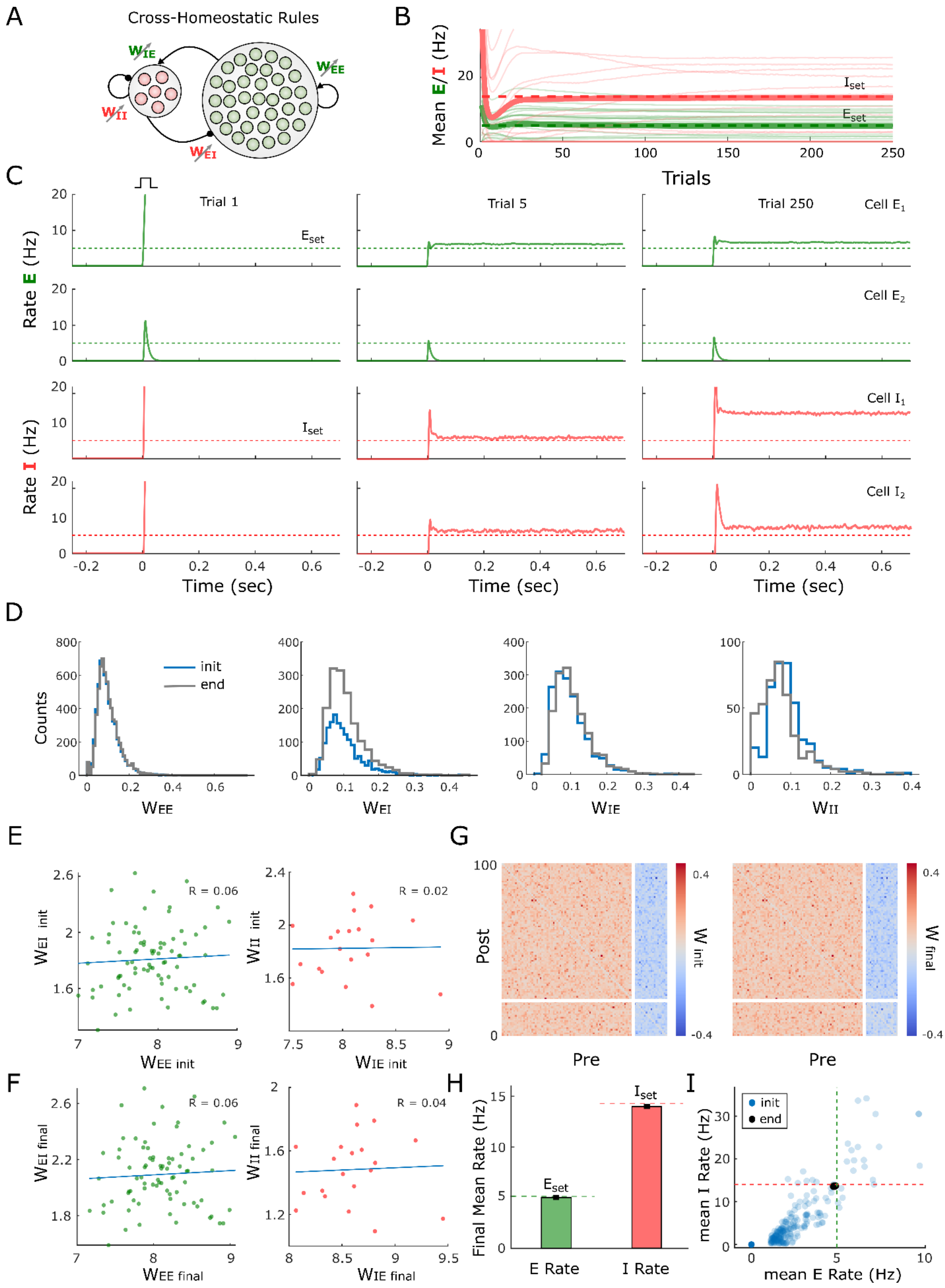
A
Cross-Homeostatic Rules
B
Mean E/I (Hz)
Trials
$I_{set}$
$E_{set}$
C
Trial 1
Trial 5
Trial 250
Cell $E_1$
Cell $E_2$
Cell $I_1$
Cell $I_2$
Rate E (Hz)
Rate I (Hz)
Time (sec)
D
Counts
init
end
$W_{EE}$
$W_{EI}$
$W_{IE}$
$W_{II}$
E
R = 0.06
R = 0.02
$W_{EE}$ init
$W_{IE}$ init
$W_{EI}$ init
$W_{II}$ init
F
R = 0.06
R = 0.04
$W_{EE}$ final
$W_{IE}$ final
$W_{EI}$ final
$W_{II}$ final
G
Post
Pre
W init
W final
H
Final Mean Rate (Hz)
E Rate
I Rate
I
mean I Rate (Hz)
mean E Rate (Hz)

**Figure S5. Log-normal initialization of weights also leads to convergence of the cross-homeostatic rules in a multi-unit model.**

**(A)** Schematic (left) of the multi-unit rate model. The network is composed of 80 excitatory and 20 inhibitory units recurrently connected. The four weight classes are governed by cross-homeostatic plasticity rules (right). See Methods for a detailed explanation of the implementation.

**(B)** Evolution of the average rate across trials of 20 excitatory and inhibitory units in an example simulation. The network is initialized with random log-normal weights (see Methods) and so neurons present diverse initial rates. $E_{set}$=5 and $I_{set}$=14 represent the target homeostatic setpoints. Red and green lines represent the individual (thin lines) and average (thick lines) firing rate of inhibitory and excitatory population, respectively.

**(C)** Example of the firing rate of two excitatory and two inhibitory units at different points in **B**. The evolution of the firing rate of the excitatory and inhibitory population within a trial in response to a brief external input is shown in every plot. Individual units converge to stable self-sustained dynamics but not to the defined setpoint.

**(D)** Initial distribution of weights at the beginning (blue) and end of the simulation (grey).

**(E)** E-I weight relationships at the beginning of the simulation. Every dot represents the total presynaptic weight onto a single unit. Left excitatory neurons. Right inhibitory neurons.

**(F)** Same plot as in **E** at the end of the simulation.

**(G)** Weight matrix for the multi-unit model at the beginning (left) and end (right) of the simulation. Inhibitory weights are shown in blue, and excitatory weights in red.

**(H)** Average firing rate of the units of the multi-unit model and for different initializations of weights (n=400). The network converges to the setpoints in average. Data represents mean ± SEM.

**(I)** Same data as in **H** but showing the average initial rate of the network for the multiple initializations (blue dots) and the average rate at the end (black). Target rates are shown in dotted lines (green, $E_{set}$=5, red $I_{set}$=14).

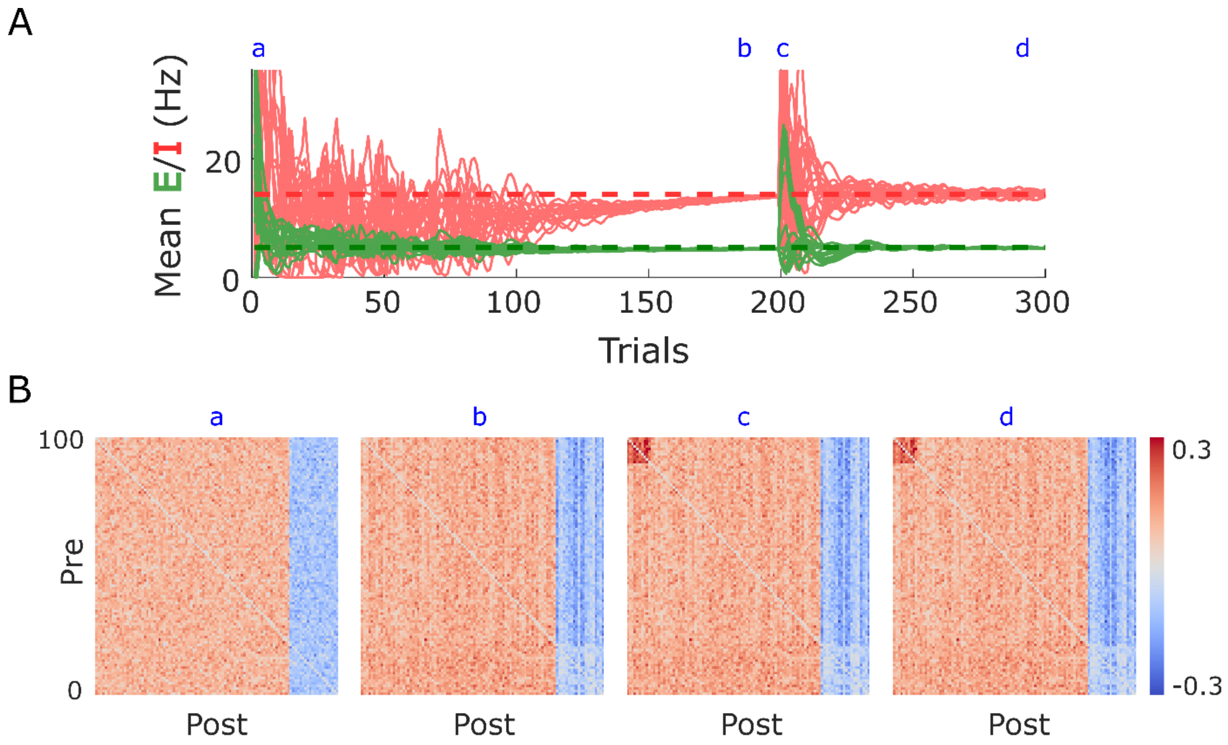


**Figure S6. Hebbian-like changes in the connectivity matrix are preserved in the presence of two-term cross-homeostatic plasticity.**

**(A)** Same example as in **Fig. 6B**, where a Hebbian change in the connectivity matrix is introduced at Trial 200. A ‘memory’ is imprinted in the first 10 excitatory neurons by increasing their recurrent weights by a constant factor. This change drives the network away from its setpoints and reengages the two-term cross-homeostatic rules. The rules successfully bring the network rates back to the setpoints, while preserving much of the differential connectivity between the altered weights. Setpoints are shown in dashed lines, $E_{set}$=5 and $I_{set}$=14.

**(B)** Weight matrix of the network at four different time points labeled in **A**. a) Initial random weight matrix. b) The weight matrix after two-term cross homeostatic plasticity has driven the network rates to setpoints. c) A Hebbian-like change in connectivity is imposed into the recurrent weights of the first 10 excitatory neurons. d) Weight matrix after two-term cross-homeostatic plasticity re-stabilizes the firing rates. Note that the ‘memory’ imposed into the weight matrix is preserved.

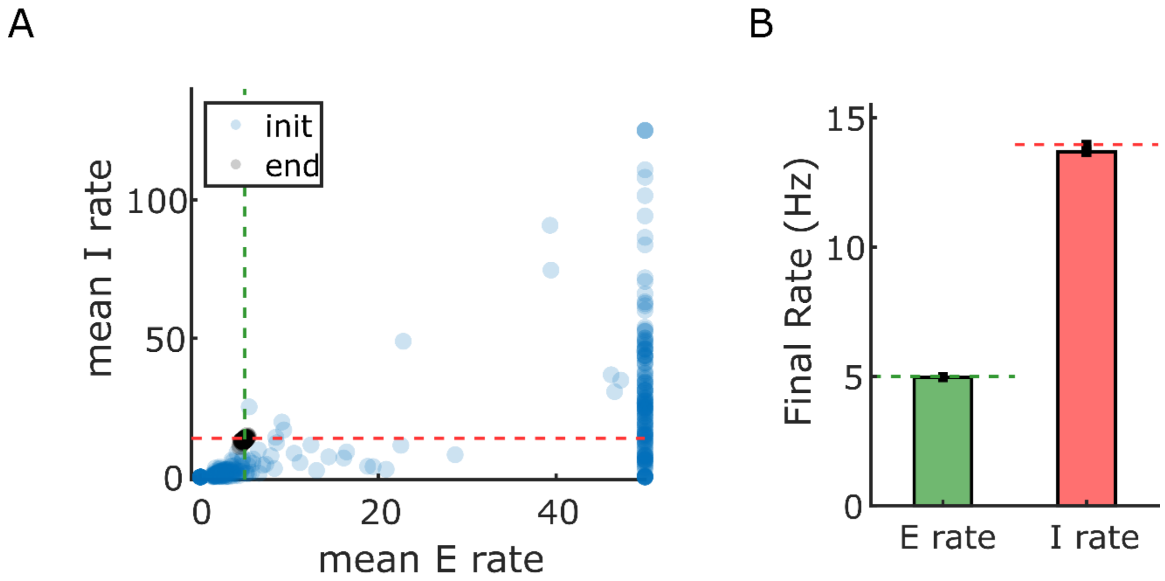


**Figure S7. Broader weight initializations for the multi-unit model also lead to convergence of the cross-homeostatic rules.**

**(A)** Average initial rate of the network for multiple weight initializations (blue dots) and the average rate at the end (black dots) after cross-homeostatic plasticity. Target setpoints are shown in dotted lines (green, $E_{set}$=5, red $I_{set}$=14). Networks have the same parameters as in **Fig.5**. Mean weights are initialized as $W_{EE}$[0,10], $W_{EI}$[0,10], $W_{IE}$[0,10], $W_{II}$[0,10], with a normally distributed standard deviation of 10% around the means (n=400).

**(B)** Same simulations as in **A** showing the final average firing rate of the units of the multi-unit model for the different networks. The networks converge to the setpoints on average. Data represents mean ± SEM.

## Supplementary Methods

### *Numerical simulations of the firing rate model*

For all simulations, the weights were updated after the completion of every trial. The trials lasted 2 seconds. Note that the value of $E$ and $I$ on every rule are implemented as average firing rates. The average of $E$ and $I$ is computed after every trial and then is low pass filtered by a process with a time constant $\tau_{trial} = 2$. The numerical integration time step was 0.1 ms. A minimum weight of 0.1 was set for all weights.

A saturation to the excitatory and inhibitory firing rate (100 and 250 Hz, respectively) was added to prevent the nonbiological scenario in which activity could diverge towards infinity under unstable conditions. Note that at the fixed point the saturation is not necessary for the cross-homeostatic rule because it is inherently stable as proved in the Supplementary Material (**Section 1.3**).

In **Fig. 2D** and **4D-G** we initialize the weights uniformly between the following ranges: $W_{EE}$[4,7], $W_{EI}$[0.5,2], $W_{IE}$[7,13], $W_{II}$[0.5,2]. Simulations were run for 3000 trials to assess stability and convergence. Note that this initialization was chosen for visualization purposes in order to represent a range of initial values surrounding the E-I balance line attractor, but the rules are robust to much broader and equal initializations for all weights, $W_{EE}$[0,12], $W_{EI}$[0,12], $W_{IE}$[0,12], $W_{II}$[0,12], (**Supplementary Figure S3**). We emphasize that many of these initializations resulted in starting conditions with exploding network rates, which were held in check by the saturation imposed on the transfer function. Despite this initial instability, the rules successfully brought the rates to the setpoint values.

### *Analytical stability analyses of the firing rate model*

We analyzed the entire dynamical system (composed of the neural subsystem and the learning rule subsystem) for every synaptic learning rule considered in this work, and analyzed its stability. In every case, the general prescription is:

a) Take the combined neural and learning rule subsystems and nondimensionalize all variables, so that the two different time scales are evident (fast neural, slow synaptic plasticity). For the description of the learning rule subsystem we switch from discrete-time dynamics to continuous-time dynamics: $\Delta W \rightarrow \tau_0 \, dW/dt$
b) Make a quasi-steady state (QSS) approximation of the neural subsystem. This means we will consider the neural subsystem is fast enough so that it converges “instantaneously” (when compared to the synaptic plasticity subsystem) to its corresponding fixed point. For this we will require that the stability conditions of the neural subsystem are satisfied (see below).

c) Find the steady-state solution of the synaptic plasticity subsystem, i.e. the self-sustained activity fixed point; compute the Jacobian of the synaptic plasticity subsystem at the fixed point; compute the eigenvalues of the Jacobian. Two out of the four eigenvalues are expected to be zero because the solution is not an isolated fixed point of the system but a continuous 2D plane in 4D weight space.
d) Address (linear) stability. If both nonzero eigenvalues have negative real parts, then the fixed point is stable under the learning rule; if at least one of the nonzero eigenvalues has positive real part, then the fixed point is unstable. (A note on abuse of notation: we might say indistinctly "the fixed point is stable/unstable" and "the learning rule is stable/unstable".)

For a detailed explanation see **Section 2** in the Supplementary Material.

### *Implementation of the Multi-unit firing rate model*

A rate-based recurrent network model containing $N_e$ = 80 excitatory and $N_i$ = 20 inhibitory neurons was implemented with all-to-all connectivity (without self-connections). The activation of the neurons followed equations (1), (2) and (3) in the main Methods. The same parameters as for the population model were used, where $W_{XY}$ represents now a matrix of synaptic weights from population $X$ to population $Y$. A minimum weight of $0.1/N_x$ for $W_{EI}$ and $W_{IE}$ and $0.1/(N_x\text{-}1)$ for $W_{EE}$ and $W_{II}$ was set for all weights.

The synaptic plasticity rules were implemented as follows.

*Cross-homeostatic family of rules:*

$$(10)\ \Delta W_{ij}^{EE} = +\alpha E_j \sum_{k=1}^{N_I} (I_{set} - I_k)/N_I$$

$$\Delta W_{ij}^{EI} = -\alpha I_j \sum_{k=1}^{N_I} (I_{set} - I_k)/N_I$$

$$\Delta W_{ij}^{IE} = -\alpha E_j \sum_{k=1}^{N_E} (E_{set} - E_k)/N_E$$

$$\Delta W_{ij}^{II} = +\alpha I_j \sum_{k=1}^{N_E} (E_{set} - E_k)/N_E\ ,$$

where $i$ and $j$ represent the post- and presynaptic neurons, respectively, and $k$ denotes the presynaptic inhibitory neurons targeting the excitatory neurons (or the

presynaptic excitatory neurons targeting an inhibitory neuron). $N_E$ and $N_I$ denote the total number of excitatory and inhibitory neurons, respectively. The weights are therefore updated following the *average* presynaptic error of the crossed E/I population classes. Note as stated above that this formulation can be implemented in a local manner (see Discussion). A learning rate of $\alpha = 0.00002$ was used for all simulations.

*Two-term cross-homeostatic family of rules:*

$$(11)\ \Delta W_{ij}^{EE} = +\alpha E_j(E_{set} - E_i) + \alpha E_j \sum_{k=1}^{N_I} (I_{set} - I_k)/N_I$$

$$\Delta W_{ij}^{EI} = -\alpha I_j(E_{set} - E_i) - \alpha I_j \sum_{k=1}^{N_I} (I_{set} - I_k)/N_I$$

$$\Delta W_{ij}^{IE} = +\alpha E_j(I_{set} - I_i) - \alpha E_j \sum_{k=1}^{N_E} (E_{set} - E_k)/N_E$$

$$\Delta W_{ij}^{II} = -\alpha I_j(I_{set} - I_i) + \alpha I_j \sum_{k=1}^{N_E} (E_{set} - E_k)/N_E\ ,$$

here the first term represents the standard homeostatic rule, and the second term cross-homeostatic plasticity (as implemented above). A learning rate of $\alpha = 0.00001$ was used for all simulations.

In **Fig. 5G-H** and **6G-H** we initialize the mean weights of the population uniformly in between the following ranges: $W_{EE}$[1,6], $W_{EI}$[0.5,2], $W_{IE}$[5,7], $W_{II}$[0.5,2]. The weights within each class were then normally distributed around that mean (and normalized by the number of neurons) with a standard deviation of 10% of the mean. Note that this initialization led to multiple initial conditions with exploding network rates (which were held in check by the saturation cutoff of the neurons). Those initial rates are not displayed in **Fig. 5-6H** for visualization purposes, but the rules successfully brought all those cases to the corresponding setpoints (final rates are displayed). Simulations were run for 1000 trials to assess stability of the convergence. Note that broader initializations for all weights $W_{EE}$[0,10], $W_{EI}$[0,10], $W_{IE}$[0,10], $W_{II}$[0,10] also result in convergence of the rules, although many more of those combinations display initial exploding network rates (**Supplementary Fig. S7)**. In the example shown in **Fig. 5A-F** and **6A-F** individual weights were normally distributed with equal mean across weights classes and standard deviation (0.1±0.04). For **Supplementary Fig. S5G-H** a lognormal distribution of weights was used. The mean weights were distributed uniformly as in Figure **5G-H**, and then weights within each class were distributed log-normally, with arithmetic standard deviation of 0.05. We note that while increasing the standard deviation of the log-normal led to more complex time-varying neural dynamics (1), the rules still converged to the average set-points.

### *Implementation of the Spiking model*

The spiking model was designed based on previous work (2). Units in the model were leaky integrate-and-fire neurons with a spike adaptation current. The membrane potential of each unit was represented as

$$(12)\ \ C_m \frac{dV(t)}{dt} = g_L(E_L - V(t)) + I_{syn}(t) - I_{adapt}(t) + \sigma\sqrt{\tau_m}\eta(t)$$

$$(13)\ \ \frac{dI_{adapt}(t)}{dt} = \frac{-I_{adapt}(t)}{\tau_{adapt}}.$$

The noise term $\sigma\sqrt{\tau_m}\eta(t)$ represents an Ornstein-Uhlenbeck process with zero mean, standard deviation $\sigma$, and a time constant equal to the membrane time constant $\tau_m = C_m/g_L$. When $V(t) \geq V_{thresh}$, the unit emitted a spike, its voltage was reset to $V_{reset}$, and its adaptation current $I_{adapt}$ was incremented by $\beta/\tau_{adapt}$. After spiking, the unit entered an absolute refractory period $\tau_{refractory}$. Default values for unit parameters can be found in **Table S1**.

Synapses were current-based, and the total synaptic current $I_{syn}(t)$ was summed across each unit's incoming synapses with distinct synaptic weights determined by the matrices $W_{EE}$, $W_{IE}$, $W_{EI}$, and $W_{II}$. Total synaptic current to a postsynaptic excitatory or inhibitory unit was given by each of the following two equations, respectively

$$(13)\ \ I_{syn}(x,t) = \sum_{y=1}^{N_{exc}} W_{EE}(x,y)\, s_{syn}(x,y,t) + \sum_{y=1}^{N_{inh}} W_{EI}(x,y)\, s_{syn}(x,y,t)$$

$$(14)\ \ I_{syn}(x,t) = \sum_{y=1}^{N_{exc}} W_{IE}(x,y)\, s_{syn}(x,y,t) + \sum_{y=1}^{N_{inh}} W_{II}(x,y)\, s_{syn}(x,y,t)\ .$$

The kinetics of the synaptic currents were determined by the function $s_{syn}(x,y,t)$ for each presynaptic unit y and postsynaptic unit x. When a presynaptic spike occurred in unit y at time $t^*$, $s_{syn}(x,y,t)$ was incremented by an amount described by a delayed difference of exponentials equation (3)

$$(15)\ \Delta s_{syn}(x,y,t) = \frac{\tau_m}{\tau_d - \tau_r}\left[\exp\left(-\frac{t-\tau_l-t^*}{\tau_d}\right) - \exp\left(-\frac{t-\tau_l-t^*}{\tau_r}\right)\right],$$

where $\tau_m$ indicates the postsynaptic membrane time constant. Thus, synaptic kinetics were determined by the delay $\tau_l$, the rise time $\tau_r$, and the decay time $\tau_d$.

The synaptic delay $\tau_l$ was uniformly distributed between 0 and 1 ms across all excitatory (inhibitory) synapses. Synaptic parameters can be found in **Table S2**.

Networks consisted of 1600 *E* units and 400 *I* units with probability of connection $p_{conn} = 0.25$ and no autapses (self-connections). Connectivity was uniformly random, and weights for each synaptic class were initialized from normal distributions with a coefficient of variation equal to 0.2. For the example training session shown in **Figure 7**, the initial weights were $\overline{W_{EE}} = 80\, pA,\ \overline{W_{IE}} = 100\, pA,$ $\overline{W_{EI}} = 350\, pA,\ \overline{W_{II}} = 225\, pA$. Network simulations were evaluated using forward Euler integration using a time step of 0.1 ms.

During each trial (1.5 s) of training a brief external current large enough to cause a spike ($I_{syn} \Rightarrow I_{syn} +\ 0.98\, nA$) was injected into 100 *E* units. This constituted a “kick” (2, 4) that provided the possibility for recurrent excitation to ignite a self-sustaining Up-state. After each trial, the contiguous time period of nonzero FR was detected, and each unit’s FR during that time period was calculated. FRs for each unit in each trial contributed to a moving average vector with a time constant of 2 trials, which we refer to as $\vec{r}_{exc}$ and $\vec{r}_{inh}$. Accordingly, we refer to the firing rate setpoints as $r_{excSet}$ and $r_{inhSet}$, which were set to 5 and 14 Hz respectively, just as in the firing rate model.

The combined plasticity equations used in the spiking model were each formulated as a sum of homeostatic (first) and local cross-homeostatic (second) terms

$$
\begin{aligned}
(16)\ \ \Delta W_{EE} &= +\alpha \cdot \vec{r}_{exc}^{\,\mathrm{T}} \cdot (r_{excSet} - \vec{r}_{exc}) + \alpha \cdot \vec{r}_{exc}^{\,\mathrm{T}} \cdot (r_{inhSet} - \vec{r}_{inhCross}) \\
\Delta W_{EI} &= -\alpha \cdot \vec{r}_{inh}^{\,\mathrm{T}} \cdot (r_{excSet} - \vec{r}_{exc}) - \alpha \cdot \vec{r}_{inh}^{\,\mathrm{T}} \cdot (r_{inhSet} - \vec{r}_{inhCross}) \\
\Delta W_{IE} &= +\alpha \cdot \vec{r}_{exc}^{\,\mathrm{T}} \cdot (r_{inhSet} - \vec{r}_{inh}) - \alpha \cdot \vec{r}_{exc}^{\,\mathrm{T}} \cdot (r_{excSet} - \vec{r}_{excCross}) \\
\Delta W_{II} &= -\alpha \cdot \vec{r}_{inh}^{\,\mathrm{T}} \cdot (r_{inhSet} - \vec{r}_{inh}) + \alpha \cdot \vec{r}_{inh}^{\,\mathrm{T}} \cdot (r_{excSet} - \vec{r}_{excCross})\,,
\end{aligned}
$$

where $\alpha$ is a learning rate constant set to $0.0025\frac{pA}{Hz^2}$. For the local cross-homeostatic term, each element of $\vec{r}_{popCross}$ represents the average FR of the units in the opposite population that synapse onto that unit. This is calculated by multiplying the unit FRs by the connectivity matrix $A_{XY}$ and dividing by the vector that results from summing its columns, which we refer to as $\vec{a}_{XY}$. Note that the $\oslash$ symbol refers to element-wise division

$$
\begin{aligned}
(17)\ \vec{r}_{inhCross} &= A_{EI}\vec{r}_{inh} \oslash \vec{a}_{EI} \\
\vec{r}_{excCross} &= A_{IE}\vec{r}_{exc} \oslash \vec{a}_{IE}\,.
\end{aligned}
$$

For a vector of presynaptic FRs ($\vec{r}_{exc}$, $\vec{r}_{inh}$) we imposed a minimum value such that each element was at least 1 Hz (otherwise networks can get stuck in the down-state). Additionally, all synaptic weights were bounded to stay within minimum and maximum weight values of 10 pA and 750 pA respectively.

For the paradoxical effect analysis in **Figure 7I**, the adaptation current was disabled for all units in order to allow for a long and stable Up-state. In each trial, a kick was given at 100 ms. From 3 to 4 s, a small positive current was injected into all inhibitory units. 40 trials were conducted at each value of the injected current, and a PSTH for each value was constructed using the inhibitory population spiking activity across all trials.

For the analysis in **Figure 7L**, nine networks were trained from distinct mean weight values for the four synaptic classes as shown in **Table S3**. We measured the error of unit firing rates with respect to their setpoints at the beginning and end of training, as quantified by the mean-squared error (MSE) across all individual excitatory and inhibitory units at each trial

$$(18)\ \ MSE(trial) = \frac{1}{2000}\sum_{i=1}^{2000}\left(\vec{r}_i - r_{popSet}\right)^2,$$

where $\vec{r}_i$ represented the FR of a unit in that population (excitatory or inhibitory) and $r_{popSet}$ represented the corresponding set-point.

**Table S1. Unit parameters**

| **Cell Parameter** | **Symbol** | **Value (E)** | **Value (I)** | **Unit** |
| --- | --- | --- | --- | --- |
| Resting potential | $E_L$ | 7.6 | 6.5 | mV |
| Reset potential | $V_{reset}$ | 14 | 14 | mV |
| Spike threshold | $V_{thresh}$ | 20 | 20 | mV |
| Refractory period | $\tau_{refractory}$ | 5 | 2 | ms |
| Membrane capacitance | $C_m$ | 200 | 100 | pF |
| Leak conductance | $g_L$ | 10 | 10 | nS |
| Membrane time constant | $\tau$ | 20 | 10 | ms |
| Adaptation strength | $\beta$ | 3 | 0 | nA·ms |
| Adaptation time constant | $\tau_a$ | 500 | n/a | ms |
| Noise standard deviation | $\sigma$ | 2.5 | 2.5 | mV |

Model parameters defining intrinsic properties of excitatory (E) and inhibitory (I) units.

**Table S2. Synaptic parameters**

| Synaptic Parameter | Symbol | Value (E) | Value (I) | Unit |
|---|---|---|---|---|
| Rise time | $\tau_r$ | 8 | 1 | ms |
| Fall time | $\tau_d$ | 23 | 1 | ms |
| Mean synaptic delay | $\tau_l$ | 1 | 0.5 | ms |

Model parameters defining kinetics of excitatory (E) and inhibitory (I) synapses.

**Table S3. Initial weight means for robustness analysis**

| Network | $\overline{W_{EE}}$ | $\overline{W_{IE}}$ | $\overline{W_{EI}}$ | $\overline{W_{II}}$ |
|---|---|---|---|---|
| 1 | 50 | 75 | 300 | 300 |
| 2 | 100 | 75 | 400 | 300 |
| 3 | 125 | 75 | 500 | 300 |
| 4 | 75 | 100 | 300 | 200 |
| 5 | 100 | 100 | 200 | 200 |
| 6 | 125 | 100 | 300 | 200 |
| 7 | 75 | 100 | 300 | 100 |
| 8 | 100 | 125 | 200 | 100 |
| 9 | 125 | 125 | 100 | 100 |

Initial means for each synaptic class in robustness analysis (all in units of pA).

## REFERENCES


1. Khajeh R, Fumarola F, & Abbott L (2022) Sparse balance: Excitatory-inhibitory networks with small bias currents and broadly distributed synaptic weights. *PLoS computational biology* 18(2):e1008836.
2. Jercog D, *et al.* (2017) UP-DOWN cortical dynamics reflect state transitions in a bistable network. *eLife*.
3. Brunel N & Wang X-J (2003) What determines the frequency of fast network oscillations with irregular neural discharges? I. Synaptic dynamics and excitation-inhibition balance. *Journal of neurophysiology* 90(1):415-430.
4. DeWeese MR & Zador AM (2006) Non-Gaussian membrane potential dynamics imply sparse, synchronous activity in auditory cortex. *J. Neurosci.* 26(47):12206-12218.

Supplementary Material

# Paradoxical Self-sustained Dynamics Emerge from Orchestrated Excitatory and Inhibitory Homeostatic Plasticity Rules

Soldado-Magraner, Seay, Laje & Buonomano 2022

June 30, 2022

## Contents


**1 Summary of results** **1**
- 1.1 *Homeostatic* plasticity . . . . . . . . . 1
- 1.2 Homeo-antiHomeo variations . . . . . 2
- 1.3 *Cross-Homeostatic* plasticity . . . . . . 3
- 1.4 *Two-Term* plasticity . . . . . . . . . . 3
- 1.5 *SynapticScaling* plasticity . . . . . . . 5
- 1.6 *ForcedBalance* plasticity . . . . . . . . 6

**2 Detailed calculations** **7**
- 2.1 Overview . . . . . . . . . . . . . . . . 7
- 2.2 Neural dynamics . . . . . . . . . . . . 8
- 2.3 *Homeostatic* plasticity: Detailed calculation . . . . . . . . . . . . . . . . . . 10
- 2.4 Detailed calculations for the other rules 13
- 2.5 Stability of the rules in a non-paradoxical regime . . . . . . . . . . . 13

**3 Derivation of a plasticity rule from a loss function** **14**
- 3.1 General prescription . . . . . . . . . . 14
- 3.2 Detailed calculation . . . . . . . . . . 15


## 1 Summary of results

In this section we describe the general results of the analytical stability analyses of the joint neural and synaptic plasticity subsystems. We express results in terms of the "free weights" $W_{EE}$ and $W_{IE}$. Subscript "up" is used to identify values at the nontrivial fixed point where $E$ and $I$ are larger than zero (as opposed to "down" where $E = I = 0$ which is the other possible solution). In Section 2 we provide a detailed description of the approach.

### 1.1 *Homeostatic* plasticity

In continuous-time dynamics, the equations for the Homeostatic plasticity rule are

$$\begin{aligned}
\frac{dW_{EE}}{dt} &= +\alpha_{EE}\, E(E_{set} - E)\\
\frac{dW_{EI}}{dt} &= -\alpha_{EI}\, I(E_{set} - E)\\
\frac{dW_{IE}}{dt} &= +\alpha_{IE}\, E(I_{set} - I)\\
\frac{dW_{II}}{dt} &= -\alpha_{II}\, I(I_{set} - I)
\end{aligned} \tag{1}$$

The condition for the fixed point to be stable (i.e., the two nonzero eigenvalues to have negative real parts, see Section 2) under this rule is:

$$\begin{aligned}
&(E_{set}^2\alpha_{IE} + I_{set}^2\alpha_{II})I_{set}(W_{EEup}g_E - 1) <\\
&(E_{set}^2\alpha_{EE} + I_{set}^2\alpha_{EI})(E_{set}W_{IEup}g_E - \Theta_I g_E)
\end{aligned} \tag{2}$$

It is difficult to determine whether the stability condition of Eq. 2 is satisfied for a general set of parameter values (see numerical analysis below). However, this condition can be re-expressed in a more useful form in terms of $W_{EE}$ and $W_{II}$:

$$\begin{aligned}
(R^2\alpha_3+\alpha_4)&(W_{EEup}\, g_E - 1)g_I\\
&< (R^2 + \alpha_2)(W_{IIup}\, g_I + 1)g_E
\end{aligned} \tag{3}$$

where

$$\begin{aligned}
R &= E_{set}/I_{set}\\
\alpha_2 &= \alpha_{EI}/\alpha_{EE}\\
\alpha_3 &= \alpha_{IE}/\alpha_{EE}\\
\alpha_4 &= \alpha_{II}/\alpha_{EE}
\end{aligned}$$

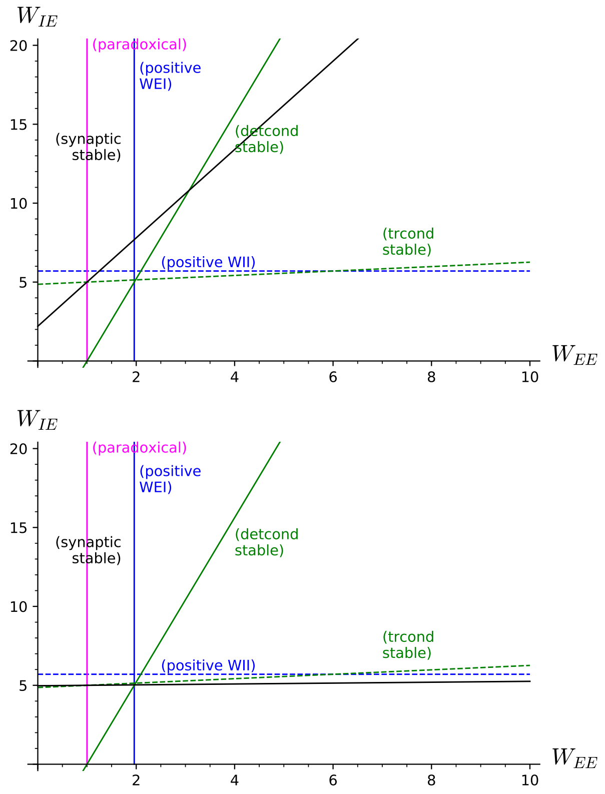


Figure S8: Regions of stability, *Homeostatic* plasticity. (Top) For biologically backed parameter values (Table 1) and learning rates of the same value ($\alpha_{XY} = 0.02$), the stability region of the Homeostatic plasticity (left of black line) has little overlap with the region where the neural subsystem is stable (triangle between the two green lines in the top-left quadrant). (Bottom) Setting $\alpha_{EE} = \alpha_{EI} = 0.02$ and $\alpha_{IE} = \alpha_{II} = 0.0002$ enlarges the stability region of the plasticity rule and makes it overlap with the stability region of the neural subsystem. Every label is on the side where the corresponding condition holds (synaptic stable: Eq. 2; detcond stable: Eq. 22; trcond stable: Eq. 23; positive $W_{EI}$: Eq. 25; positive $W_{II}$: Eq. 26; paradoxical: Eq. 27).

Note that learning rate values of the same order lead to $\alpha_{2,3,4} \sim 1$ and that biologically backed parameter values satisfy:

$$I_{set} > E_{set}$$

$$g_I > g_E$$

both likely preventing the condition to hold. On the other hand, if $\alpha_{IE}$ and $\alpha_{II}$ are small enough (slow dynamics of the weights onto the inhibitory neuron) the rule can be stable. See the step-by-step derivation of this stability condition in Section 2.3.

As an illustration of the results above, in Figure S8(top) we plot the stability condition Eq. 2 with parameter values as in Table 1 and learning rates $\alpha_{XY} = 0.02$. It is clear that the plasticity rule is stable in a region with little overlap with the stability region of the neural subsystem. The stability region can be enlarged by making the dynamics of the weights onto the inhibitory neuron slower, as in Figure S8(bottom) where $\alpha_{EE} = \alpha_{EI} = 0.02$ and $\alpha_{IE} = \alpha_{II} = 0.0002$.

See Section 2.3 for a detailed analysis.

## 1.2 Homeo-antiHomeo variations

The stability condition in the previous section was obtained by assuming all learning rates are positive. Interestingly, if some of them are negative then the fixed point may still be stable. A negative learning rate can be interpreted as the corresponding equation being *anti*-homeostatic, i.e. if the neural activity ($E$ or $I$) departs from its setpoint then the rule will drive it even farther away. While this kind of behavior would be usually deemed undesired, it is worth considering due to its relationship with the paradoxical regime.

In this section we consider the Homeostatic rule, Eq. 28, and let the learning rates $\alpha_{XY}$ be either positive or negative. The particular case where all learning rates are positive corresponds to the original Homeostatic plasticity rule.

Once we free the signs of the learning rates, the fixed point needs two conditions to be stable:

$$(R^2\alpha_3+\alpha_4)(W_{EE\,up}\, g_E - 1)g_I < (R^2 + \alpha_2)(W_{II\,up}\, g_I + 1)g_E \quad (4)$$

$$(R^2\alpha_3+\alpha_4)(R^2 + \alpha_2) > 0 \quad (5)$$

where

$$R = E_{set}/I_{set}$$
$$\alpha_2 = \alpha_{EI}/\alpha_{EE}$$
$$\alpha_3 = \alpha_{IE}/\alpha_{EE}$$
$$\alpha_4 = \alpha_{II}/\alpha_{EE}$$

Eq. 4 is equal to the stability condition of the original Homeostatic rule (Eq. 3). The additional condition Eq. 5 is very interesting in that it allows the fixed point to be stable, for instance, under full anti-Homeo plasticity where all four learning rates are negative (leading to $\alpha_{2,3,4}$ all positive).

See details in the corresponding section of the SageMath-Jupyter notebook:
`upstates-Homeostatic stability.ipynb`

## 1.3 *Cross-Homeostatic* plasticity

In continuous-time dynamics, the equations for the Cross-Homeostatic plasticity rule are

$$\begin{aligned} \frac{dW_{EE}}{dt} &= +\alpha_{EE} E(I_{set} - I) \\ \frac{dW_{EI}}{dt} &= -\alpha_{EI} I(I_{set} - I) \\ \frac{dW_{IE}}{dt} &= -\alpha_{IE} E(E_{set} - E) \\ \frac{dW_{II}}{dt} &= +\alpha_{II} I(E_{set} - E) \end{aligned} \tag{6}$$

and its stability condition in terms of the free weights $W_{EE}$ and $W_{IE}$ reads:

$$\begin{aligned} &(E_{set}^2 \alpha_{EE} + I_{set}^2 \alpha_{EI}) I_{set} W_{IEup} g_E \\ &> -(E_{set}^2 \alpha_{IE} + I_{set}^2 \alpha_{II}) \\ &\quad ((W_{EEup} g_E - 1) E_{set} - \Theta_E g_E) \end{aligned} \tag{7}$$

This stability condition can be put in a simpler form by switching to $W_{EI}$ and $W_{IE}$:

$$(R^2 \alpha_3 + \alpha_4) W_{EIup} + (R^2 + \alpha_2) W_{IEup} > 0 \tag{8}$$

(where $R$ and $\alpha_{2,3,4}$ are defined as in the previous subsection). This condition is always satisfied because the weights and parameters are positive definite and thus the rule is stable for any choice of parameter values (as long as the neural subsystem is). Fig. S9 shows the stability region of the neural subsystem for the set of parameter values of Table 1. Any choice of values for the weights $W_{EE}$ and $W_{IE}$ within the stability region of the neural subsystem will lead to a stable fixed point.

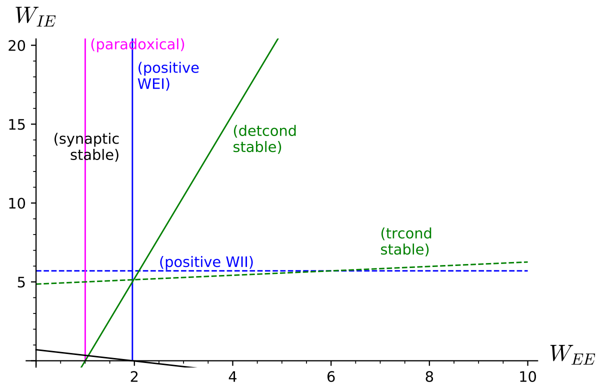


Figure S9: Stability of the *Cross-Homeostatic* rule. The rule is stable for any parameter value; the fixed point is thus stable where the neural subsystem is stable, i.e. in the upper right region between the two green lines. Every label is on the side where the corresponding condition holds (synaptic stable: Eq. 7; detcond stable: Eq. 22; trcond stable: Eq. 23; positive $W_{EI}$: Eq. 25; positive $W_{II}$: Eq. 26; paradoxical: Eq. 27). Parameter values as in Table 1.

See Section 2.4 for a detailed analysis.

## 1.4 *Two-Term* plasticity

The equations for the Two-Term plasticity rule in continuous-time dynamics are

$$\begin{aligned} \frac{dW_{EE}}{dt} &= +\alpha E(I_{set} - I) + \beta E(E_{set} - E) \\ \frac{dW_{EI}}{dt} &= -\alpha I(I_{set} - I) - \beta I(E_{set} - E) \\ \frac{dW_{IE}}{dt} &= -\alpha E(E_{set} - E) + \beta E(I_{set} - I) \\ \frac{dW_{II}}{dt} &= +\alpha I(E_{set} - E) - \beta I(I_{set} - I) \end{aligned} \tag{9}$$

and its stability condition in terms of the free weights

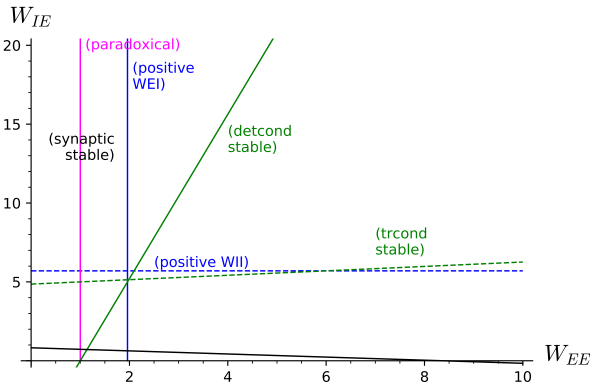


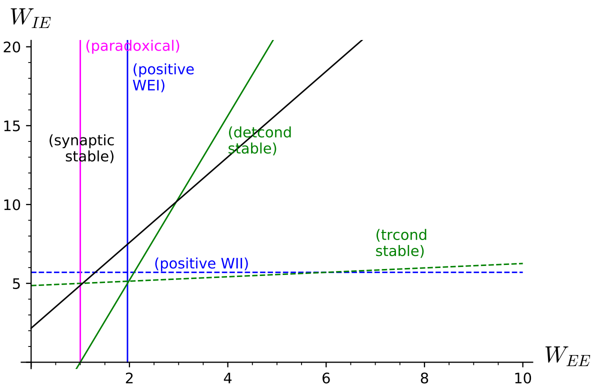


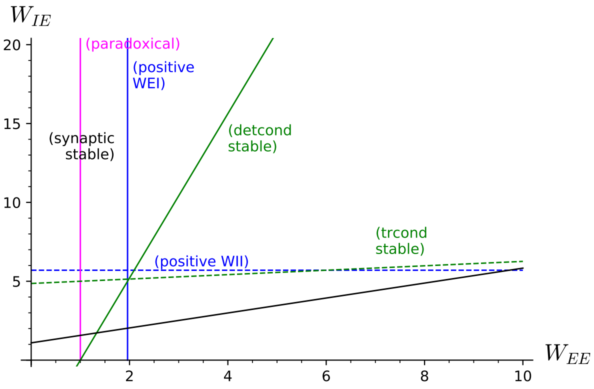


Figure S10: Regions of stability, *Two-Term* rule. (Top Left) $\alpha = 0.02$, $\beta = 0.005$. (Top Right) $\alpha = 0.02$, $\beta = 0.02$. (Bottom Left) $\alpha = 0.0002$, $\beta = 0.02$. Every label is on the side where the corresponding condition holds (synaptic stable: Eq. 10; detcond stable: Eq. 22; trcond stable: Eq. 23; positive $W_{EI}$: Eq. 25; positive $W_{II}$: Eq. 26; paradoxical: Eq. 27). Parameter values as in Table 1.

$W_{EE}$ and $W_{IE}$ is

$$\begin{aligned}(I_{set}\alpha + E_{set}\beta)W_{IEup}g_E \\ > (I_{set}\beta - E_{set}\alpha)W_{EEup}g_E \\ + (\Theta_E g_E + E_{set})\alpha + (\Theta_I g_E - I_{set})\beta\end{aligned} \tag{10}$$

In Figure S10 we plot the stability condition of this rule, Eq. 10, for three different parameter values: $\alpha \gg \beta$ (the "Cross-Homeostatic" terms dominate over the "Homeostatic" terms, and the rule is stable with the largest stability region); $\alpha = \beta$ (the two terms are of comparable size); and $\alpha \ll \beta$ (the "Homeostatic" terms dominate instead, and the stability region of the rule is as small as that of the Homeostatic plasticity).

In order to determine the validity of the stability condition, Eq. 10, in a more general situation, we rewrite it in a more useful form:

$$(a - b)\beta < (a' + b' + c)\alpha \tag{11}$$

where

$$\begin{aligned}a &= (W_{EEup}g_E - 1)E_{set}I_{set}g_I \\ a' &= (W_{EEup}g_E - 1)E_{set}^2 g_I \\ b &= (W_{IIup}g_I + 1)E_{set}I_{set}g_E \\ b' &= (W_{IIup}g_I + 1)I_{set}^2 g_E \\ c &= (I_{set}\Theta_I - E_{set}\Theta_E)g_E g_I\end{aligned}$$

Note that the following is satisfied for a biologically backed set of parameter values:

$$\begin{aligned}I_{set} > E_{set} \\ \Theta_I > \Theta_E\end{aligned}$$

and thus it is likely that $c > 0$. In addition, $b$ and $b'$ are positive definite, and $a, a' > 0$ in the paradoxical regime ($W_{EE}g_E - 1 > 0$). All this makes the stability condition likely satisfied, and thus the plasticity rule stable. Finally, a small enough $\beta$ would make the condition more likely to hold.

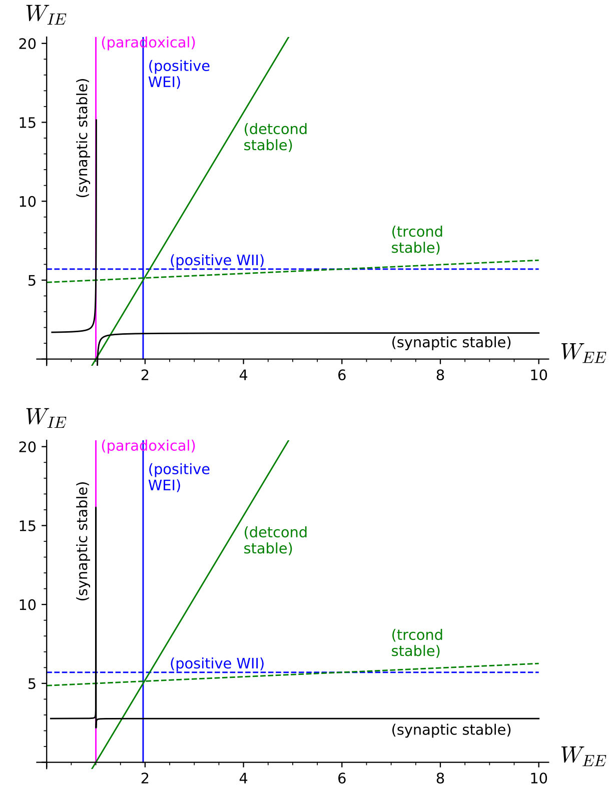

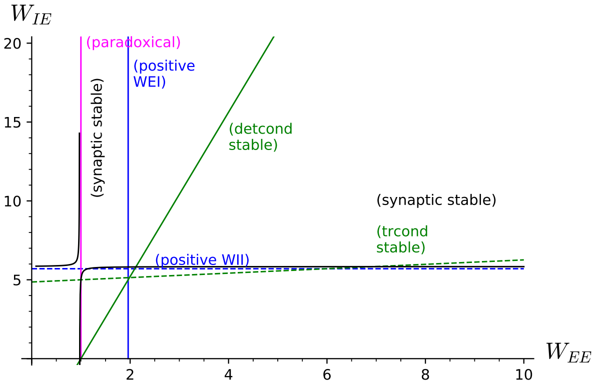


Figure S11: Regions of stability, *SynapticScaling* rule. (Top Left) Equal rates ($\alpha_{XY} = 0.02$). (Top Right) Slow inhibitory ($\alpha_{EE,EI} = 0.02$, $\alpha_{IE,II} = 0.002$). (Bottom Left) Slow excitatory ($\alpha_{EE,EI} = 0.002$, $\alpha_{IE,II} = 0.02$). Every label is on the side where the corresponding condition holds (synaptic stable: Eq. 13 after switching to $W_{EE}$ and $W_{IE}$; detcond stable: Eq. 22; trcond stable: Eq. 23; positive $W_{EI}$: Eq. 25; positive $W_{II}$: Eq. 26; paradoxical: Eq. 27). Parameter values as in Table 1.

See Section 2.4 for a detailed analysis.

## 1.5 ***SynapticScaling* plasticity**

The equations for the SynapticScaling plasticity rule in continuous-time dynamics are

$$\begin{aligned}\frac{dW_{EE}}{dt} &= +\alpha_{EE}(E_{set} - E)W_{EE}\\ \frac{dW_{EI}}{dt} &= -\alpha_{EI}(E_{set} - E)W_{EI}\\ \frac{dW_{IE}}{dt} &= +\alpha_{IE}(I_{set} - I)W_{IE}\\ \frac{dW_{II}}{dt} &= -\alpha_{II}(I_{set} - I)W_{II}\end{aligned} \qquad (12)$$

and the condition for the fixed point to be stable under this rule is

$$(W_{EE\,up}g_E - 1)a < (W_{II\,up}g_I + 1)b \qquad (13)$$

where

$$\begin{aligned}a &= (I_{set}W_{II}\alpha_4 + \Theta_I\alpha_3)g_I\\ b &= E_{set}W_{EE\,up}g_E\\ &\quad + ((W_{EE\,up}g_E - 1)E_{set} - \Theta_E g_E)\alpha_2\\ &\quad - (W_{EE\,up}g_E - 1)I_{set}\alpha_3\end{aligned}$$

(where $\alpha_{2,3,4}$ are defined as in previous subsections). This stability condition does not hold for biologically backed parameter values unless the dynamics of the weights onto the inhibitory neuron are slow enough (and in a few fine-tuned cases). To show this, we express the stability condition in terms of the free weights $W_{EE}$ and $W_{IE}$ and plot it with parameter values as in Table 1 and equal rates ($\alpha_{XY} = 0.02$; Figure S11 top left). The stability condition is a homographic function (i.e. a hyperbola) with stability regions in its upper-left and lower-right quadrants—entirely outside the stability region of the neural sub-

system. If the dynamics of the weights onto the excitatory neuron are made slower, the homographic function is even steeper (bottom left); if the weights onto the inhibitory neuron are made slower instead, the stability regions switch and overlap with the stability region of the neural subsystem, making the fixed point stable (top right).

It is illustrative to consider the particular case where all learning rates are equal. In this case the stability condition, Eq. 13, doesn't depend on the learning rates and takes the simpler form:

$$\begin{aligned}(W_{IIup}g_I{+}1)a > {} & (W_{EEup}g_E - 1)a' \\ & + (W_{EEup}g_E - 1)(W_{IIup}g_I + 1)b\end{aligned} \tag{14}$$

where

$$\begin{aligned}a &= (E_{set}W_{EEup} - \Theta_E)g_E \\ a' &= (I_{set}W_{IIup} + \Theta_I)g_I \\ b &= I_{set} - E_{set}\end{aligned}$$

Note that in a biologically backed set of parameter values the following is true:

$$\begin{aligned}I_{set} &> E_{set} \\ g_I &> g_E \\ \Theta_I &> \Theta_E\end{aligned}$$

This makes $b > 0$ and likely $a' > a$ (in addition, $a'$ is a sum of positive terms while $a$ is a difference). Then in the paradoxical regime ($W_{EE}g_E - 1 > 0$) it seems likely that the stability condition is not satisfied, because the right-hand side is a sum of positive terms and one of them is likely greater than the left-hand side. The SynapticScaling rule is then likely unstable when the learning rates are equal.

A more general case with different learning rates can be analyzed by grouping terms in the following way:

$$\begin{aligned}(I_{set}W_{IIup}\alpha_4 + \Theta_I\alpha_3)g_I(W_{EEup}g_E - 1)& \\ <(((W_{EEup}g_E - 1)E_{set} - \Theta_E g_E)\alpha_2& \\ - (W_{EEup}g_E - 1)I_{set}\alpha_3& \\ + E_{set}W_{EEup}g_E)(W_{IIup}g_I + 1)&\end{aligned}$$

If $(W_{EE}g_E - 1) > 0$ (paradoxical regime), then decreasing $\alpha_3$ and/or $\alpha_4$ (slow dynamics of the weights onto the inhibitory neuron) helps satisfying the condition and thus making the rule stable.

See Section 2.4 for a detailed analysis.

## 1.6 *ForcedBalance* plasticity

The equations for the ForcedBalance plasticity rule are

$$\begin{aligned}\frac{dW_{EE}}{dt} &= +\alpha_1 g_E E(E_{set} - E) \\ \frac{dW_{EI}}{dt} &= \frac{1}{\tau_0}(W_{EIup} - W_{EI}) \\ \frac{dW_{IE}}{dt} &= +\alpha_3 g_I E(I_{set} - I) \\ \frac{dW_{II}}{dt} &= \frac{1}{\tau_0}(W_{IIup} - W_{II})\end{aligned} \tag{15}$$

and the conditions for the fixed point to be stable under this rule are

$$\begin{aligned}a_1 + b_1(W_{IIup}\, g_I + 1) &< b_1'(W_{EEup}\, g_E - 1) \\ a_2 + b_2(W_{IIup}\, g_I + 1) &< b_2'(W_{EEup}\, g_E - 1)\end{aligned} \tag{16}$$

where

$$\begin{aligned}a_1 &= (I_{set}\Theta_E\Theta_I\,\alpha_1\, g_E g_I + E_{set}^3\alpha_3)\, g_E g_I \\ b_1 &= I_{set}^2\Theta_E\,\alpha_1 g_E^2 g_I - E_{set}^2 I_{set}\,\alpha_1\, g_E^2 \\ b_1' &= E_{set}I_{set}\Theta_I\,\alpha_1\, g_E g_I^2 + E_{set}^2 I_{set}\,\alpha_3\, g_I^2 \\ a_2 &= 2\Theta_E\Theta_I\,\alpha_1\, g_E^2 g_I^2 \\ b_2 &= 2I_{set}\Theta_E\,\alpha_1\, g_E^2 g_I - E_{set}^2\,\alpha_1\, g_E^2 \\ b_2' &= 2E_{set}\Theta_I\,\alpha_1\, g_E g_I^2 + E_{set}^2\,\alpha_3\, g_I^2\end{aligned}$$

In Figure S12 we plot the stability condition of this rule, Eq. 16, for three different parameter values: $\alpha_1 = \alpha_3$, $\alpha_1 \gg \alpha_3$ (inhibitory plasticity slower); and $\alpha_1 \ll \alpha_3$ (excitatory plasticity slower).

In order to decide whether conditions Eq. 16 are satisfied in a more general case, note that $b_1$ and $b_2$ on the left-hand side are subtractions whereas $b_1'$ and $b_2'$ on the right-hand side are sums of positive definite terms, which helps satisfying the condition. On the other hand, one of the stability conditions of the neural subsystem might counter the effect: $(W_{IIup}\, g_I + 1)\tau_E > (W_{EEup}\, g_E - 1)\tau_I$ (see Section 2.2 below) but for biologically backed parameter values it is $\tau_E > \tau_I$ thus leaving room for the condition to hold. See Section 2.4 for a detailed analysis.

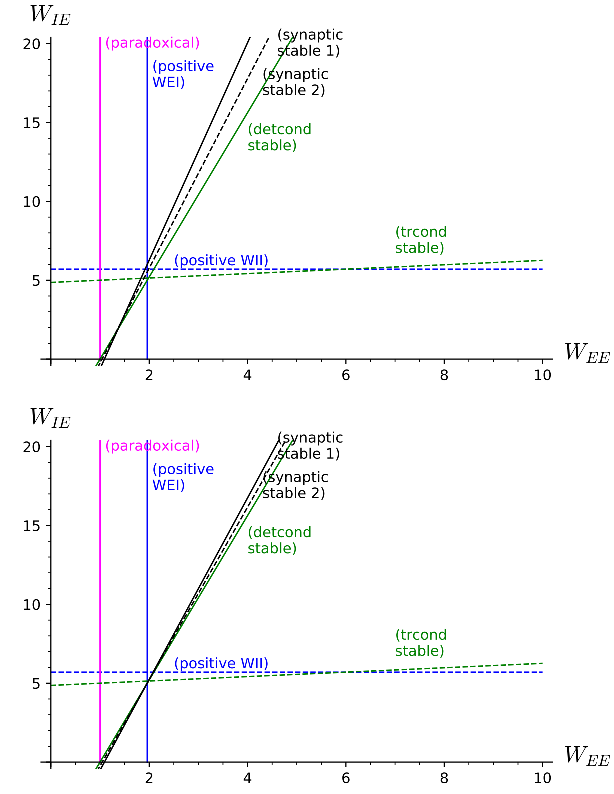


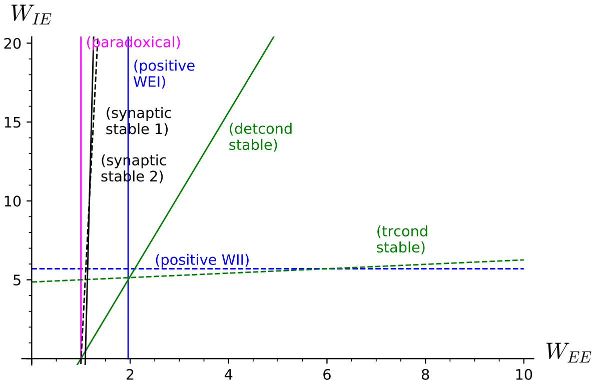


Figure S12: Regions of stability, *ForcedBalance* rule. (Top Left) Equal rates ($\alpha_{1,3} = 0.02$). (Top Right) Slow inhibitory ($\alpha_1 = 0.02$, $\alpha_3 = 0.0002$). (Bottom Left) Slow excitatory ($\alpha_1 = 0.0002$, $\alpha_3 = 0.02$). Every label is on the side where the corresponding condition holds (synaptic stable 1 and 2: Eq. 16 after switching to $W_{EE}$ and $W_{IE}$; detcond stable: Eq. 22; trcond stable: Eq. 23; positive $W_{EI}$: Eq. 25; positive $W_{II}$: Eq. 26; paradoxical: Eq. 27). Parameter values as in Table 1.

# 2 Detailed calculations

## 2.1 Overview

We analyze the whole neural+synaptic system for every synaptic plasticity rule considered in this work, and study their stability. In every case, the general prescription is:

1. Take the combined neural+synaptic system and nondimensionalize all variables [see Sections 1.2 and 1.4 of Ref. 1][see Section 3.5 of Ref. 2], so that the two different time scales are evident (fast neural, slow synaptic).

2. Make a quasi-steady state (QSS) approximation of the neural subsystem [1, 2]. This means we will consider the neural subsystem is fast enough so that it converges "instantaneously" (when compared to the synaptic subsystem) to its corresponding fixed point. For this we will require that the stability conditions of the neural subsystem are satisfied (see below).

3. Find the steady-state solution of the synaptic subsystem, i.e. the fixed point; compute the Jacobian of the synaptic subsystem at the fixed point; compute the eigenvalues of the Jacobian [2, 3]. Two out of the four eigenvalues are expected to be zero because the fixed point is not an isolated fixed point of the system but a continuous 2D plane in 4D weight space.

4. Address (linear) stability. If both nonzero eigenvalues have negative real part, then the fixed point is stable under the plasticity rule; if at least one of the nonzero eigenvalues has positive real part, then the fixed point is unstable [2, 3]. (A note on abuse of notation: we might say indis-

tinctly "the fixed point is stable/unstable" and "the plasticity rule is stable/unstable".)

Eigenvalues and stability in the presence of continuous, i.e. non-isolated, attractors have been discussed in the context of neural networks for eye position control [4, 5] (keep in mind that the eigenvalues' critical value in these references is 1 instead of zero because they consider eigenvalues of the connectivity matrix alone, whereas we consider eigenvalues of the whole linear part). As the fixed point is a collection of non-isolated fixed points that form a 2D plane, there is no dynamics along the plane, and the linear stability analysis is enough to fully address stability—we do have two zero eigenvalues, but there is no need to compute the center manifold [3] because the other two eigenvalues represent the whole dynamics around the fixed point and have nonzero real part.

In order to apply the tools from Dynamical Systems' theory for flows in a unified way for both the neural and synaptic subsystems, we will switch from a discrete-time description of synaptic weight dynamics (where the change in weight $W$ is represented by $\Delta W$ applied every certain time interval) to a continuous-time description (where the weights are continuously evolving albeit with a long time scale $\tau_0$):

$$\Delta W \to \tau_0 \frac{dW}{dt}$$

In the following we first define the neural subsystem and compute its stability conditions (next subsection). Then we consider every plasticity rule in detail (following subsections).

**Paradoxical regime.** In this text we show detailed calculations of the stability conditions for the Homeostatic plasticity in the paradoxical regime only; see Section 2.5 for the non-paradoxical case.

## 2.2 Neural dynamics

For the neural+synaptic system in the QSS approximation to be stable under a specific synaptic plasticity rule, it is necessary that the neural subsystem is stable so it remains in its QSS solution as the weights evolve. In this section we define the neural subsystem and compute its stability conditions.

(SageMath code in the Supplementary Material: `upstates-Neural subsystem stability.ipynb`)

### 2.2.1 System's equations and fixed points

We consider a two-subpopulation model with firing-rate units $E$ and $I$ with ReLU activation functions (gain $g_X$, threshold $\Theta_X$, with $X = E, I$). The dynamics for synaptic currents above threshold is given by:

$$\begin{aligned} \frac{dE}{dt} &= \frac{1}{\tau_E}(-E + g_E(W_{EE}E - W_{EI}I - \Theta_E)) \\ \frac{dI}{dt} &= \frac{1}{\tau_I}(-I + g_I(W_{IE}E - W_{II}I - \Theta_I)) \end{aligned} \tag{17}$$

All variables and parameters are definite positive. In this subsection the synaptic weights $W_{XY}$ are fixed.

**Neural fixed point.** The fixed point of the neural subsystem in the supreathreshold regime is the solution of $dE/dt = dI/dt = 0$:

$$\begin{aligned} E_{up} &= (W_{EI}\, g_I\, \Theta_I - (W_{II}\, g_I + 1)\, \Theta_E)\, g_E/C \\ I_{up} &= ((W_{EE}\, g_E - 1)\, \Theta_I - W_{IE}\, g_E\, \Theta_E)\, g_I/C \end{aligned} \tag{18}$$

where

$$C = W_{EI}W_{IE}\, g_E\, g_I - (W_{II}\, g_I + 1)(W_{EE}\, g_E - 1) \tag{19}$$

We named it with the subscript "up" to distinguish it from the trivial solution "down" where $E$ and $I$ are zero (and the neural subsystem is below threshold).

The activity of the excitatory and inhibitory subpopulations at the nontrivial fixed point, $E_{up}$ and $I_{up}$, depend on all weight values. Only some of the combinations, however, lead to a stable steady state. We compute the stability conditions in the following subsection.

### 2.2.2 Stability of the nontrivial neural fixed point

The Jacobian matrix, that is the matrix of first derivatives, gives information regarding the stability

of fixed points: if the real parts of its eigenvalues are all negative, then the fixed point is stable.

The Jacobian of the neural system (Eq. 17) is

$$J = \begin{pmatrix} (W_{EE} g_E - 1)/\tau_E & -W_{EI} g_E/\tau_E \\ W_{IE} g_I/\tau_I & -(W_{II} g_I + 1)/\tau_I \end{pmatrix} \tag{20}$$

Its eigenvalues can be expressed as:

$$\lambda_{1,2} = \frac{1}{2}\left(Tr \pm \sqrt{Tr^2 - 4Det}\right) \tag{21}$$

where $Tr$ and $Det$ are the trace and determinant of the matrix, respectively. For eigenvalues either complex or purely real, their real parts are negative (and thus the fixed point is stable) when $Det > 0$ and $Tr < 0$, that is:

$$W_{EI} W_{IE} g_E g_I > (W_{EE} g_E - 1)(W_{II} g_I + 1) \tag{22}$$

$$(W_{II} g_I + 1)\tau_E > (W_{EE} g_E - 1)\tau_I \tag{23}$$

Note that the positive determinant condition, Eq. 22, is equivalent to $C > 0$ (Eq. 19).

In the following, we will require that the stability conditions of the neural subsystem, Eqs. 22 and 23, are satisfied.

### 2.2.3 Weight values consistent with the neural fixed point

The fixed point relationships, Eq. 18, are expressed as the $E$ and $I$ values resulting from a given set of weight values. If we set instead $E$ and $I$ to their target values $E_{set}$ and $I_{set}$ and solve for the weights, we get the weight values that are consistent with a given fixed point activity:

$$\begin{aligned} W_{EIup} &= \frac{(E_{set} W_{EEup} - \Theta_E)\, g_E - E_{set}}{I_{set}\, g_E} \\ W_{IIup} &= \frac{(E_{set} W_{IEup} - \Theta_I)\, g_I - I_{set}}{I_{set}\, g_I} \end{aligned} \tag{24}$$

Note first that any stable plasticity rule for the evolution of the weights for the neural subsystem (Eq. 17) must converge to weight values in accordance with these relationships (either in the form Eq. 24 or Eq. 18).

Second, note that the system is underdetermined and that is why two of the weights are free (chosen to be $W_{EE}$ and $W_{EI}$). Note also that all weight values must be positive; specifically, requiring $W_{EIup} > 0$ and $W_{IIup} > 0$ leads to

$$W_{EEup} > \frac{\Theta_E\, g_E + E_{set}}{E_{set}\, g_E} \tag{25}$$

$$W_{IEup} > \frac{\Theta_I\, g_I + I_{set}}{E_{set}\, g_I} \tag{26}$$

We refer to these expressions as the "positive $W_{EI}$" and the "positive $W_{II}$" conditions, respectively.

### 2.2.4 Paradoxical effect

The paradoxical effect arises when an external depolarization of the inhibitory subpopulation (increase of $I$) produces an actual *decrease* of $I$. In this model, an external depolarization of $I$ can be mimicked by a decrease of its threshold $\Theta_I$, thus there is a paradoxical effect whenever the coefficient of $\Theta_I$ in the numerator of $I_{up}$ is positive. The coefficient is $g_I\,(W_{EE}\,g_E - 1)/C$ and thus there is paradoxical effect if

$$W_{EE}\, g_E - 1 > 0 \tag{27}$$

The paradoxical effect can also be seen in a plot of the fixed point values $E_{up}$ and $I_{up}$ (Eq. 18) as a function of each individual weight. Specifically, from a naive point of view $I_{up}$ should increase when $W_{IE}$ is increased, and decrease when $W_{II}$ is increased; however, it does the opposite in either case (see Figure S13).

| | | | | | |
|---|---|---|---|---|---|
| $I_{set}$ | = | 14 | $E_{set}$ | = | 5 |
| $g_I$ | = | 4 | $g_E$ | = | 1 |
| $\Theta_I$ | = | 25 | $\Theta_E$ | = | 4.8 |
| $\tau_I$ | = | 2 | $\tau_E$ | = | 10 |

Table 1: Parameter values throughout the Supplementary Material. This set of parameter values makes the neural subsystem to be in the paradoxical regime (i.e. the fixed point is an inhibition-stabilized fixed point [6]). For non-paradoxical conditions, see Section 2.5.

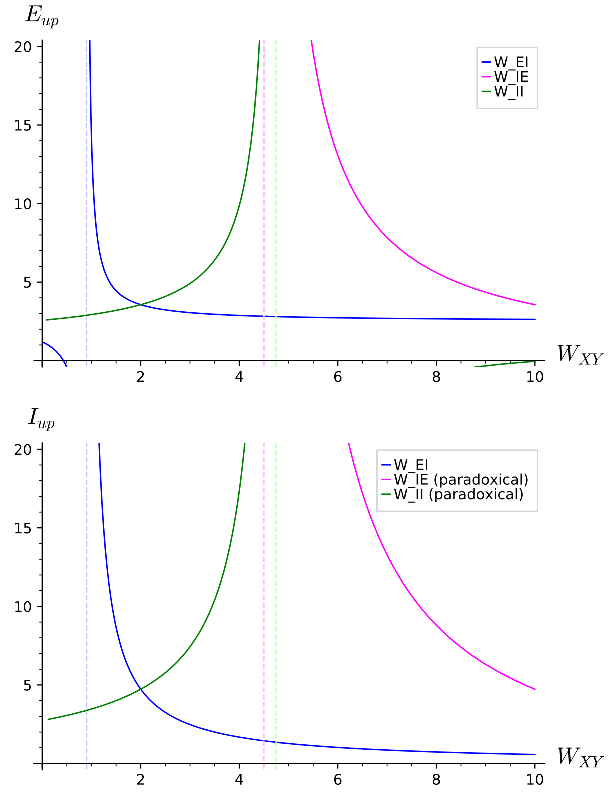


Figure S13: Paradoxical effect in the neural subsystem ($W_{EE} = 5$; parameter values as in Table 1). $E_{up}$ behaves as expected when each weight is varied. $I_{up}$, however, shows paradoxical behavior when either $W_{IE}$ or $W_{II}$ are varied. Dashed lines are the vertical asymptote of every case.

## 2.3 *Homeostatic* plasticity: Detailed calculation

In this section we show in detail the calculation of the stability condition for the Homeostatic plasticity rule.

(SageMath code in the Supplementary Material: `upstates-Homeostatic stability.ipynb`)

### 2.3.1 Definition of the plasticity rule

In continuous-time dynamics, the Homeostatic plasticity rule reads:

$$\begin{aligned}\frac{dW_{EE}}{dt} &= +\alpha_{EE}\, E(E_{set} - E)\\ \frac{dW_{EI}}{dt} &= -\alpha_{EI}\, I(E_{set} - E)\\ \frac{dW_{IE}}{dt} &= +\alpha_{IE}\, E(I_{set} - I)\\ \frac{dW_{II}}{dt} &= -\alpha_{II}\, I(I_{set} - I)\end{aligned} \tag{28}$$

where $\alpha_{XY}$ $(X, Y = E, I)$ are the learning rates (with appropriate units) setting the time scales of the weight dynamics, and $E_{set}$ and $I_{set}$ are the set points of the excitatory and inhibitory subpopulations, respectively.

The fixed points of the system (i.e. steady states) are determined by setting all derivatives to zero. There is a non-trivial fixed point compatible with the neural subsystem being above threshold: it is the set of weight values such that:

$$\begin{aligned}E_{up} &= E_{set}\\ I_{up} &= I_{set}\end{aligned} \tag{29}$$

The values of the weights corresponding to the nontrivial neural fixed point are given by the (underdetermined) system defined by equating Eqs. 29 and 18. Since it is a two-equation system for a set of four unknown weights, there are two free weights that we choose to be $W_{EEup}$ and $W_{IEup}$. The values of the other two are given by Eq. 24. This means that the fixed point is actually a continuous set of non-isolated fixed points forming a 2D plane in 4D weight space. In other words, there is an infinite number of weight values compatible with the nontrivial neural fixed point (possibly not all stable, though).

### 2.3.2 Nondimensionalization

Next we nondimensionalize all variables in order to have a simpler system and make the QSS approximation in a safe way. We define new (nondimensional) variables $e$, $i$, $\tau$, $w_{EE}$, $w_{EI}$, $w_{IE}$, and $w_{II}$, and their

corresponding scaling parameters. We substitute the new variables into the full system (neural+synaptic, Eqs. 17 and 28) and choose the values of the scaling parameters such that all nondimensional variables are of order 1 (see attached SageMath code). With this, the full system reads:

$$\begin{aligned}
\epsilon_E \frac{de}{d\tau} &= -e + Rew_{EE} - \frac{iw_{EI}}{R} - \theta_E \\
\epsilon_I \frac{di}{d\tau} &= -i + \frac{Rew_{IE}}{g} - \frac{iw_{II}}{Rg} - \theta_I \\
\frac{dw_{EE}}{d\tau} &= -e(e-1) \\
\frac{dw_{EI}}{d\tau} &= +\alpha_2 i(e-1) \\
\frac{dw_{IE}}{d\tau} &= -\alpha_3 e(i-1) \\
\frac{dw_{II}}{d\tau} &= +\alpha_4 i(i-1)
\end{aligned} \tag{30}$$

where we defined the new parameters

$$\begin{aligned}
\epsilon_E &= \tau_E/\tau_0 \\
\epsilon_I &= \tau_I/\tau_0 \\
\tau_0 &= 1/(\alpha g_E E_{set} I_{set}) \\
R &= E_{set}/I_{set} \\
g &= g_E/g_I \\
\alpha_2 &= \alpha_{EI}/\alpha_{EE} \\
\alpha_3 &= \alpha_{IE}/\alpha_{EE} \\
\alpha_4 &= \alpha_{II}/\alpha_{EE} \\
\theta_E &= (g_E/E_{set})\Theta_E \\
\theta_I &= (g_I/I_{set})\Theta_I
\end{aligned}$$

#### 2.3.3 Quasi-steady state approximation

Neural dynamics evolves in a much shorter time scale ($\tau_E$ and $\tau_I$) than synaptic dynamics ($\tau_0$):

$$\begin{aligned}
\tau_E \ll \tau_0 &\implies \epsilon_E \ll 1 \\
\tau_I \ll \tau_0 &\implies \epsilon_I \ll 1
\end{aligned}$$

which implies

$$\begin{aligned}
\epsilon_E \frac{de}{d\tau} &\sim 0 \\
\epsilon_I \frac{di}{d\tau} &\sim 0
\end{aligned} \tag{31}$$

thus we can safely assume $e$ and $i$ very quickly reach quasi-equilibrium values, i.e. practically instantaneous convergence to quasi-steady state (QSS) values as if the weights were fixed, while the synaptic weights evolve according to their slow dynamics. This allows us to reduce the system's dimensionality from six to four.

In the QSS approximation, the values of the nondimensionalized excitatory and inhibitory activities instantaneously track the slow dynamics of the plasticity rule. They are determined by applying Eq. 31 to the first two rows of Eq. 30; solving for $e$ and $i$ leads to

$$\begin{aligned}
e_{qss} &= (g\theta_I w_{EI} - (w_{II} + Rg)\theta_E)/c \\
i_{qss} &= (Rg\theta_I(Rw_{EE} - 1) - R^2\theta_E w_{IE})/c
\end{aligned} \tag{32}$$

where

$$c = Rw_{EI}w_{IE} - (w_{II} + Rg)(Rw_{EE} - 1)$$

The full system in the QSS approximation reads

$$\begin{aligned}
\frac{dw_{EE}}{d\tau} &= -e_{qss}(e_{qss} - 1) \\
\frac{dw_{EI}}{d\tau} &= +\alpha_2 i_{qss}(e_{qss} - 1) \\
\frac{dw_{IE}}{d\tau} &= -\alpha_3 e_{qss}(i_{qss} - 1) \\
\frac{dw_{II}}{d\tau} &= +\alpha_4 i_{qss}(i_{qss} - 1)
\end{aligned} \tag{33}$$

where $e_{qss}$ and $i_{qss}$ are nonlinear functions of the weights as defined by Eq. 32.

Note that the nontrivial neural fixed point, defined by making all derivatives equal to zero, can be expressed as

$$\begin{aligned}
e_{qss} &= 1 \\
i_{qss} &= 1
\end{aligned} \tag{34}$$

which is the nondimensionalized version of Eq. 29. The weight values compatible with this condition are defined by equating Eqs. 32 and 34:

$$\begin{aligned}
w_{EIup} &= R(Rw_{EEup} - 1) - R\theta_E \\
w_{IIup} &= R(Rw_{IEup} - g) - Rg\theta_I
\end{aligned} \tag{35}$$

($w_{EEup}$ and $w_{IEup}$ are free). This is the nondimensionalized version of Eq. 24.

### 2.3.4 Stability condition

The program for assessing linear stability of the fixed point is as follows: a) compute the Jacobian (the matrix of first derivatives) of Eq. 33 and evaluate it at the fixed point; b) compute the eigenvalues of the Jacobian (two of them will be zero because the fixed points form a continuous 2D plane in phase space); c) If the real part of the two nonzero eigenvalues is negative then the fixed point is stable; if at least one of the nonzero eigenvalue has positive real part then the fixed point is unstable.

**Jacobian matrix.** Let the full system in the QSS approximation (Eq. 33) be written as

$$\frac{dw_{EE}}{d\tau} = f_{EE}(e_{qss}, i_{qss})$$
$$\frac{dw_{EI}}{d\tau} = f_{EI}(e_{qss}, i_{qss})$$
$$\text{etc}\ldots$$

where $e_{qss}$ and $i_{qss}$ are functions of the weights as defined by Eq. 32. By applying the chain rule the elements $J_{ij}$ $(i, j = 1 \ldots 4)$ of the Jacobian matrix can be expressed as

$$J_{11} = \frac{df_{EE}}{dw_{EE}} = \frac{df_{EE}}{de_{qss}}\frac{de_{qss}}{dw_{EE}} + \frac{df_{EE}}{di_{qss}}\frac{di_{qss}}{dw_{EE}}$$
$$J_{12} = \frac{df_{EE}}{dw_{EI}} = \frac{df_{EE}}{de_{qss}}\frac{de_{qss}}{dw_{EI}} + \frac{df_{EE}}{di_{qss}}\frac{di_{qss}}{dw_{EI}}$$
$$J_{13} = \ldots$$
$$J_{21} = \frac{df_{EI}}{dw_{EE}} = \frac{df_{EI}}{de_{qss}}\frac{de_{qss}}{dw_{EE}} + \frac{df_{EI}}{di_{qss}}\frac{di_{qss}}{dw_{EE}}$$
$$J_{22} = \ldots$$
$$\text{etc}\ldots$$

In order to have the Jacobian specialized in the fixed point, these expressions are to be substituted by Eqs. 32-35.

**Eigenvalues of the Jacobian matrix.** The Jacobian matrix has two zero eigenvalues and two nonzero eigenvalues. The nonzero eigenvalues have the form:

$$\lambda_\pm = \frac{A \pm \sqrt{A^2 - DC}}{C} \tag{36}$$

where

$$\begin{aligned} A &= R^2 g\theta_I + (R^2\alpha_3 + \alpha_4)Rw_{EEup} \\ &\quad - (R^2 + \alpha_2)Rw_{IEup} + \alpha_2 g\theta_I - R^2\alpha_3 - \alpha_4 \\ C &= 2R(Rg\theta_I w_{EEup} - R\theta_E w_{IEup} - g\theta_I) \\ D &= 2(R^2\alpha_3 + \alpha_4)(R^2 + \alpha_2)/R \end{aligned} \tag{37}$$

**Sign of the eigenvalues.** To determine the sign of the real part of Eq. 36, first note that the factor $D$ is positive definite. Second, $C$ must be positive because it is related to one of the stability conditions of the neural subsystem (Eq. 22, after substituting back to dimensionalized quantities). Note next that $A^2 - DC$ is less than $A^2$ (since $C$ and $D$ are positive), and thus the square root is either real and less than $|A|$ or imaginary, both cases leading to $\text{Re}(A \pm \sqrt{A^2 - DC}) < 0$ if $A < 0$. The plasticity rule is then stable (both eigenvalues have negative real part) if $A < 0$, which in terms of the original parameters and free weights $W_{EE}$ and $W_{IE}$ reads:

$$\begin{aligned} &(E_{set}^2\alpha_{IE} + I_{set}^2\alpha_{II})I_{set}(W_{EEup}g_E - 1) < \\ &(E_{set}^2\alpha_{EE} + I_{set}^2\alpha_{EI})(E_{set}W_{IEup}g_E - \Theta_I g_E) \end{aligned} \tag{38}$$

### 2.3.5 Analysis of the stability condition

It is hard to determine whether the stability condition Eq. 38 is satisfied for a general set of parameter values (see numerical analysis below). However, by using the fixed point relationship Eq. 24, this condition can be re-expressed in a more useful form in terms of $W_{EE}$ and $W_{II}$:

$$\begin{aligned} &(R^2\alpha_3 + \alpha_4)(W_{EEup}\, g_E - 1)g_I \\ &\qquad < (R^2 + \alpha_2)(W_{IIup}\, g_I + 1)g_E \end{aligned} \tag{39}$$

Note that learning rates values of the same order lead to $\alpha_{2,3,4} \sim 1$ and that biologically backed parameter values satisfy:

$$I_{set} > E_{set}$$
$$g_I > g_E$$

both likely preventing the condition to hold.

On the other hand, small enough values of $\alpha_3$ and $\alpha_4$ (by making the dynamics of the weights onto the inhibitory neuron $W_{IE}$ and $W_{II}$ slower) would help satisfy the condition thus making the system stable.

#### 2.3.6 Relationship between the synaptic stability and the paradoxical condition

The boundary of the stability condition for this plasticity rule, Eq. 38, is a linear function in the $(W_{EE}, W_{IE})$ space with a slope that tends to infinity as the excitatory learning rates $(\alpha_{EE,EI})$ tend to zero:

$$\text{slope} = \frac{(E_{set}^2 \alpha_{IE} + I_{set}^2 \alpha_{II}) I_{set}}{(E_{set}^2 \alpha_{EE} + I_{set}^2 \alpha_{EI}) E_{set}}$$

while its root is a complicated expression (see SageMath notebook) that tends to $W_{EE} = 1/g_E$. The region of stability is to the left of the line. Thus, the boundary of stability in this limit coincides exactly with the boundary of the paradoxical condition $(W_{EE} > 1/g_E)$. This can be construed as an inconsistency/contradiction between the stability of the rule and the existence of the paradoxical effect.

## 2.4 Detailed calculations for the other rules

The stability calculations for the rest of the rules follow very similar paths. They can be found in the corresponding SageMath-Jupyter notebooks:

`upstates-CrossHomeostatic stability.ipynb`

`upstates-TwoTerm stability.ipynb`

`upstates-SynapticScaling stability.ipynb`

`upstates-ForcedBalance stability.ipynb`

## 2.5 Stability of the rules in a non-paradoxical regime

All results above were developed with the neural subsystem set in the paradoxical regime—that is, the region in $(W_{EE}, W_{IE})$ leading to a stable fixed point was completely within the paradoxical region $(W_{EE} g_E > 1)$. In order to show the importance of the paradoxical behavior for the stability of the plasticity rules, we also computed the stability conditions of every plasticity rule in a more general setting where the excitatory subpopulation in the neural subsystem has an external, constant, excitatory input current $I_{ext}$. This allows the neural subsystem to display both paradoxical and non-paradoxical stable behavior (in the second case, at the expense of the fixed point not being an inhibition-stabilized fixed point; see `upstates-Neural subsystem stability-with Iext.ipynb`).

#### 2.5.1 *Homeostatic* with $I_{ext}$

The stability condition doesn't depend on $I_{ext}$ and it reads the same as Eq. 2:

$$\begin{aligned}(E_{set}^2 \alpha_{IE} + I_{set}^2 \alpha_{II}) I_{set} (W_{EEup} g_E - 1) < \\ (E_{set}^2 \alpha_{EE} + I_{set}^2 \alpha_{EI})(E_{set} W_{IEup} g_E - \Theta_I g_E)\end{aligned} \tag{40}$$

(SageMath code in `upstates-Homeostatic stability-with Iext.ipynb`)

#### 2.5.2 *CrossHomeostatic* with $I_{ext}$

The stability condition with $I_{ext}$ is:

$$\begin{aligned}&(E_{set}^2 \alpha_{EE} + I_{set}^2 \alpha_{EI}) I_{set} W_{IEup} g_E \\ &\quad > -(E_{set}^2 \alpha_{IE} + I_{set}^2 \alpha_{II}) \\ &\qquad ((W_{EEup} g_E - 1) E_{set} - (\Theta_E - I_{ext}) g_E)\end{aligned} \tag{41}$$

which is very similar to Eq. 7 except that it has $(\Theta_E - I_{ext})$ instead of just $\Theta_E$. From this it should be evident that the condition will still hold for any positive value of $I_{ext}$ (right-hand side decreases).

The validity of the condition can also be seen after switching to $W_{IE}$ and $W_{EI}$, leading to exactly the same condition as Eq. 8:

$$(R^2 \alpha_3 + \alpha_4) W_{EIup} + (R^2 + \alpha_2) W_{IEup} > 0 \tag{42}$$

which holds for any value of $I_{ext}$.

(SageMath code in `upstates-CrossHomeostatic stability-with Iext.ipynb`)

#### 2.5.3 *TwoTerm* with $I_{ext}$

The stability condition with $I_{ext}$ is:

$$\begin{aligned}(I_{set}\alpha + E_{set}\beta)W_{IEup}g_E \\ > (I_{set}\beta - E_{set}\alpha)W_{EEup}g_E \\ + ((\Theta_E - I_{ext})g_E + E_{set})\alpha + (\Theta_I g_E - I_{set})\beta\end{aligned} \tag{43}$$

which is very similar to Eq. 10 except that it has $(\Theta_E - I_{ext})$ instead of just $\Theta_E$. From this it should be evident that the larger the value of $I_{ext}$ (right-hand side decreases) the larger the stability region.

(SageMath code in `upstates-TwoTerm stability-with Iext.ipynb`)

#### 2.5.4 *SynapticScaling* with $I_{ext}$

When $I_{ext}$ is included in the dynamics of $E$, the stability condition for the rule reads:

$$(W_{EEup}g_E - 1)a < (W_{IIup}g_I + 1)b \tag{44}$$

where

$$\begin{aligned}a &= (I_{set}W_{II}\alpha_4 + \Theta_I\alpha_3)g_I \\ b &= E_{set}W_{EEup}g_E \\ &\quad + ((W_{EEup}g_E - 1)E_{set} - (\Theta_E - I_{ext})g_E)\alpha_2 \\ &\quad - (W_{EEup}g_E - 1)I_{set}\alpha_3\end{aligned}$$

which is very similar to Eq. 13 except that it has $(\Theta_E - I_{ext})$ instead of just $\Theta_E$. From this it should be evident that including a positive $I_{ext}$ will increase the chances that the condition holds (right-hand side increases).

(SageMath code in `upstates-SynapticScaling stability-with Iext.ipynb`)

#### 2.5.5 *ForcedBalance* with $I_{ext}$

The stability conditions when $I_{ext}$ is included in the neural subsystem are:

$$\begin{aligned}a_1 + b_1(W_{IIup}\, g_I + 1) < b_1'(W_{EEup}\, g_E - 1) \\ a_2 + b_2(W_{IIup}\, g_I + 1) < b_2'(W_{EEup}\, g_E - 1)\end{aligned} \tag{45}$$

where

$$\begin{aligned}a_1 &= (I_{set}(\Theta_E - I_{ext})\Theta_I\, \alpha_1\, g_E g_I + E_{set}^3\alpha_3)\, g_E g_I \\ b_1 &= I_{set}^2(\Theta_E - I_{ext})\, \alpha_1 g_E^2 g_I - E_{set}^2 I_{set}\, \alpha_1\, g_E^2 \\ b_1' &= E_{set}I_{set}\Theta_I\, \alpha_1\, g_E g_I^2 + E_{set}^2 I_{set}\, \alpha_3\, g_I^2 \\ a_2 &= 2(\Theta_E - I_{ext})\Theta_I\, \alpha_1\, g_E^2 g_I^2 \\ b_2 &= 2I_{set}(\Theta_E - I_{ext})\, \alpha_1\, g_E^2 g_I - E_{set}^2\, \alpha_1\, g_E^2 \\ b_2' &= 2E_{set}\Theta_I\, \alpha_1\, g_E g_I^2 + E_{set}^2\, \alpha_3\, g_I^2\end{aligned}$$

which are very similar to Eqs. 16 except that there is a $(\Theta_E - I_{ext})$ instead of just $\Theta_E$. From this it should be evident that the larger the value of $I_{ext}$ (left-hand side decreases) the larger the stability region.

(SageMath code in `upstates-ForcedBalance stability-with Iext.ipynb`)

# 3 Derivation of a plasticity rule from a loss function

(SageMath code in the Supplementary Material: `upstates-Loss function.ipynb`)

Here we show how to compute a plasticity rule for the weights starting from a loss function. Then we make an approximation by considering that the weight values are close to the values corresponding to the fixed point.

## 3.1 General prescription

We consider the full neural+synaptic system in the QSS approximation (see e.g. Section 2.3). In this approximation the neural subsystem is represented by the quasi-steady-state values

$$\begin{aligned}E = E_{up}(W_{EE}, W_{EI}, W_{IE}, W_{II}) \\ I = I_{up}(W_{EE}, W_{EI}, W_{IE}, W_{II})\end{aligned} \tag{46}$$

where the functions $E_{up}$ and $I_{up}$ are defined by Eq. 18 (see [7] for a related discussion on quasi-steady state, synaptic plasticity, and gradient descent).

The synaptic subsystem, that is the plasticity rule, will be obtained as a result of considering a specific loss function, and the general prescription to compute the plasticity rule from a loss function $L$ is the following:

1. Consider a loss function depending on $E$ and $I$ (which in turn depend on all weights):

$$L = L(E, I)$$

Conditions to be satisfied by the loss function are, for instance, to be smooth enough (i.e. continuous and differentiable) and to have a minimum when the activities $E$ and $I$ are at the set points $E_{set}$ and $I_{set}$ (i.e. homeostatic plasticity).

2. The dynamics of the weights is such that it follows a gradient descent on the loss function towards its minimum. In vector notation:

$$\Delta \mathbf{W} = -\alpha \nabla L \tag{47}$$

with a single learning rate $\alpha$ for simplicity. The unfolded plasticity rules, that is the equations that govern the weights' dynamics, are then

$$\begin{aligned} \Delta W_{EE} &= -\alpha \frac{\partial L}{\partial W_{EE}} \\ \Delta W_{EI} &= -\alpha \frac{\partial L}{\partial W_{EI}} \\ \Delta W_{IE} &= -\alpha \frac{\partial L}{\partial W_{IE}} \\ \Delta W_{II} &= -\alpha \frac{\partial L}{\partial W_{II}} \end{aligned} \tag{48}$$

3. The partial derivatives of the loss function in Eq. 48 are:

$$\begin{aligned} \frac{\partial L}{\partial W_{EE}} &= \frac{\partial L}{\partial E}\frac{\partial E}{\partial W_{EE}} + \frac{\partial L}{\partial I}\frac{\partial I}{\partial W_{EE}} \\ \frac{\partial L}{\partial W_{EI}} &= \frac{\partial L}{\partial E}\frac{\partial E}{\partial W_{EI}} + \frac{\partial L}{\partial I}\frac{\partial I}{\partial W_{EI}} \\ \frac{\partial L}{\partial W_{IE}} &= \frac{\partial L}{\partial E}\frac{\partial E}{\partial W_{IE}} + \frac{\partial L}{\partial I}\frac{\partial I}{\partial W_{IE}} \\ \frac{\partial L}{\partial W_{II}} &= \frac{\partial L}{\partial E}\frac{\partial E}{\partial W_{II}} + \frac{\partial L}{\partial I}\frac{\partial I}{\partial W_{II}} \end{aligned} \tag{49}$$

or, in vector notation:

$$\nabla L = \frac{\partial L}{\partial E}\nabla E + \frac{\partial L}{\partial I}\nabla I \tag{50}$$

Here we use the chain rule for the derivatives because it gives us much more compact expressions at the end.

4. The partial derivatives in the gradients $\nabla E = \left(\frac{\partial E}{\partial W_{EE}}, \ldots\right)$ and $\nabla I = \left(\frac{\partial I}{\partial W_{EE}}, \ldots\right)$ etc. are to be taken from the quasi-steady-state values of $E$ and $I$, Eq. 46. We will, however, compute the partial derivatives from the implicit expressions given by setting $dE/dt = dI/dt = 0$ in Eq. 17 without solving for $E$ and $I$.

## 3.2 Detailed calculation

### 3.2.1 Exact plasticity rules

**Loss function.** We choose a very general loss function that depends homeostatically on both $E$ and $I$ activities:

$$L(E, I) = \frac{1}{2}(E_{set} - E)^2 + \frac{1}{2}(I_{set} - I)^2 \tag{51}$$

This loss function is an elliptic paraboloid in $(E, I)$ space with a global minimum at $(E_{set}, I_{set})$ so a gradient descend working on $E$ and $I$ should converge to that minimum (see Liapunov function and gradient systems: [3, Section 1.1B][8, Sections 9.3 and 9.4][2, Section 7.2]). Keep in mind, however, that $L$ has a different shape when expressed as a function of the weights, and that $E$ and $I$ are not necessarily monotonic functions of the weights (particularly for a paradoxical system), so the conditions for the set point of $L$ to be stable or a global minimum or even unique are not necessarily satisfied.

**Partial derivatives of $L$.** The partial derivatives of $L$ with respect to $E$ and $I$ are simply

$$\begin{aligned} \frac{\partial L}{\partial E} &= -(E_{set} - E) \\ \frac{\partial L}{\partial I} &= -(I_{set} - I) \end{aligned} \tag{52}$$

**Partial derivatives of $E$ and $I$.** We compute the partial derivatives $\partial X/\partial W_{XY}$ $(X, Y = E, I)$ by first equating the neural subsystem (Eq. 17) to zero:

$$\begin{aligned} E &= g_E(W_{EE}E - W_{EI}I - \Theta_E) \\ I &= g_I(W_{IE}E - W_{II}I - \Theta_I) \end{aligned} \tag{53}$$

then differentiating the implicit functions:

$$\begin{aligned}
\frac{\partial E}{\partial W_{EE}} &= g_E(E + W_{EE}\frac{\partial E}{\partial W_{EE}}) - g_E W_{EI}\frac{\partial I}{\partial W_{EE}} \\
\frac{\partial E}{\partial W_{EI}} &= g_E W_{EE}\frac{\partial E}{\partial W_{EI}} - g_E(I + W_{EI}\frac{\partial I}{\partial W_{EI}}) \\
\frac{\partial E}{\partial W_{IE}} &= g_E W_{EE}\frac{\partial E}{\partial W_{IE}} - g_E W_{EI}\frac{\partial I}{\partial W_{IE}} \\
\frac{\partial E}{\partial W_{II}} &= g_E W_{EE}\frac{\partial E}{\partial W_{II}} - g_E W_{EI}\frac{\partial I}{\partial W_{II}} \\
\frac{\partial I}{\partial W_{EE}} &= g_I W_{IE}\frac{\partial E}{\partial W_{EE}} - g_I W_{II}\frac{\partial I}{\partial W_{EE}} \\
\frac{\partial I}{\partial W_{EI}} &= g_I W_{IE}\frac{\partial E}{\partial W_{EI}} - g_I W_{II}\frac{\partial I}{\partial W_{EI}} \\
\frac{\partial I}{\partial W_{IE}} &= g_I(E + W_{IE}\frac{\partial E}{\partial W_{IE}}) - g_I W_{II}\frac{\partial I}{\partial W_{IE}} \\
\frac{\partial I}{\partial W_{II}} &= g_I W_{IE}\frac{\partial E}{\partial W_{II}} - g_I(I + W_{II}\frac{\partial I}{\partial W_{II}})
\end{aligned} \tag{54}$$

and then solving for the derivatives:

$$\begin{aligned}
\frac{\partial E}{\partial W_{EE}} &= -(EW_{II}\, g_E\, g_I + E g_E)/C \\
\frac{\partial E}{\partial W_{EI}} &= (IW_{II}\, g_E\, g_I + I g_E)/C \\
\frac{\partial E}{\partial W_{IE}} &= EW_{EI}\, g_E\, g_I/C \\
\frac{\partial E}{\partial W_{II}} &= -IW_{EI}\, g_E\, g_I/C \\
\frac{\partial I}{\partial W_{EE}} &= -EW_{IE}\, g_E\, g_I/C \\
\frac{\partial I}{\partial W_{EI}} &= IW_{IE}\, g_E\, g_I/C \\
\frac{\partial I}{\partial W_{IE}} &= (EW_{EE}\, g_E - E) g_I/C \\
\frac{\partial I}{\partial W_{II}} &= -(IW_{EE}\, g_E - I) g_I/C
\end{aligned} \tag{55}$$

where

$$C = W_{EI} W_{IE}\, g_E\, g_I - (W_{II}\, g_I + 1)(W_{EE}\, g_E - 1)$$

**Exact plasticity rules.** Putting everything together, the plasticity rules Eq. 48 are:

$$\begin{aligned}
\Delta W_{EE} &= -\frac{\alpha}{C}((I_{set} - I)EW_{IE}\, g_e\, g_I \\
&\quad + (E_{set} - E)E(W_{II}\, g_I + 1)g_E) \\
\Delta W_{EI} &= +\frac{\alpha}{C}((I_{set} - I)IW_{IE}\, g_e\, g_I \\
&\quad + (E_{set} - E)I(W_{II}\, g_I + 1)g_E) \\
\Delta W_{IE} &= +\frac{\alpha}{C}((E_{set} - E)EW_{EI}\, g_e\, g_I \\
&\quad + (I_{set} - I)E(W_{EE}\, g_E - 1)g_I) \\
\Delta W_{II} &= -\frac{\alpha}{C}((E_{set} - E)IW_{EI}\, g_e\, g_I \\
&\quad + (I_{set} - I)I(W_{EE}\, g_E - 1)g_I)
\end{aligned} \tag{56}$$

Note that these are very complicated, nonlinear expressions because both $E$ and $I$ depend on all weights via Eq. 53. Also the denominator $C$ depends on all weights (see previous paragraph).

### 3.2.2 Approximation

We want simpler expressions for the plasticity rules. Note that the exact expressions above all have a homeostatic factor (either $E - E_{set}$ or $I - I_{set}$) and a presynaptic factor (either $E$ or $I$), while the rest are complicated expressions coming from the derivatives $\partial E/\partial W_{XY}$ and $\partial I/\partial W_{XY}$. We want to keep the homeostatic and presynaptic factors as they are while simplifying the rest of the expressions (explicit dependence on the weights including $C$) by performing a lowest-order Taylor series expansion of the explicit dependence of Eqs. 55 on the weights. Although this is not a textbook Taylor expansion of the full expressions, it is very informative because the results can be much more easily interpreted (for a similar approach see [7]).

We perform a zeroth-order approximation of the derivatives $\partial E/\partial W_{XY}$ and $\partial I/\partial W_{XY}$ as functions of the weights (i.e. while holding the presynaptic factors $E$ and $I$ constant) around the fixed point. In this approximation the weights are not small but close to their target values, represented by the relationships Eq. 24. By substituting the result in Eq. 49, we get

the following approximated plasticity rules:

$$\begin{aligned}\Delta W_{EE} &= +\alpha_E E(I_{set} - I) + \beta_E E(E_{set} - E) \\ \Delta W_{EI} &= -\alpha_E I(I_{set} - I) - \beta_E I(E_{set} - E) \\ \Delta W_{IE} &= -\alpha_I E(E_{set} - E) + \beta_I E(I_{set} - I) \\ \Delta W_{II} &= +\alpha_I I(E_{set} - E) - \beta_I I(I_{set} - I)\end{aligned} \tag{57}$$

where

$$\begin{aligned}\alpha_E &= \alpha g_E E_{set} W_{IEup}/D \\ \alpha_I &= \alpha A/D \\ \beta_E &= \alpha g_E B/D \\ \beta_I &= \alpha I_{set}(1 - W_{EEup} g_E)/D\end{aligned}$$

and

$$\begin{aligned}A &= E_{set} W_{EEup} g_E - \Theta_E g_E - E_{set} \\ B &= E_{set} W_{IEup} - \Theta_I \\ D &= \Theta_I W_{EEup} g_E - \Theta_E W_{IEup} g_E - \Theta_I\end{aligned}$$

**Analysis.** Note that $\alpha_E$, $\alpha_I$, $\beta_E$, and $\beta_I$ are all constant. Furthermore, note that

- $A > 0$ as it is equal to the "positive $W_{EI}$" condition, Eq. 25;
- $B > 0$ as it is part of the "positive $W_{II}$" condition, Eq. 26;
- $D > 0$ as it is equal to the numerator of $I_{up}$, Eq. 18 (up to a positive factor), which must be positive because the denominator is.

Interestingly, note that the learning rate $\beta_I$ can be either negative or positive depending on whether the fixed point where the dynamics is converging to is paradoxical ($W_{EEup} g_E - 1 > 0$) or not ($W_{EEup} g_E - 1 < 0$).

Note that the terms with $\alpha_{E,I}$ in the approximated plasticity rules, Eq. 57, are exactly equal to the Cross-Homeostatic rules, Eq. 6. Additionaly, the terms with $\beta_{E,I}$ are exactly equal to the Homeostatic rules, Eq. 28, unless $\beta_I < 0$ which would make the plasticity rule a Cross-Homeo-antiHomeo hybrid.

# References


1. Keener, J. P. & Sneyd, J. *Mathematical physiology* (Springer, 1998).
2. Strogatz, S. H. *Nonlinear dynamics and chaos with student solutions manual: With applications to physics, biology, chemistry, and engineering* (CRC press, 2018).
3. Wiggins, S. *Introduction to applied nonlinear dynamical systems and applications* (Springer-Verlag, 1996).
4. Seung, H. S. How the brain keeps the eyes still. *Proceedings of the National Academy of Sciences* **93,** 13339–13344 (1996).
5. Seung, H. S. Continuous attractors and oculomotor control. *Neural Networks* **11,** 1253–1258 (1998).
6. Sadeh, S. & Clopath, C. Inhibitory stabilization and cortical computation. *Nature Reviews Neuroscience* **22,** 21–37 (2021).
7. Mackwood, O., Naumann, L. B. & Sprekeler, H. Learning excitatory-inhibitory neuronal assemblies in recurrent networks. *bioRxiv.* `https://doi.org/10.1101/2020.03.30.016352` (2020).
8. Hirsch, M. W. & Smale, S. *Differential equations, dynamical systems, and linear algebra* (Academic press, 1974).
